# Low-Expressing Synucleinopathy Mouse Models Based on Oligomer-Forming Mutations and C-Terminal Truncation of α-Synuclein

**DOI:** 10.3389/fnins.2021.643391

**Published:** 2021-06-17

**Authors:** Ana Martinez Hernandez, Ivan Silbern, Insa Geffers, Lars Tatenhorst, Stefan Becker, Henning Urlaub, Markus Zweckstetter, Christian Griesinger, Gregor Eichele

**Affiliations:** ^1^Genes and Behavior Department, Max Planck Institute for Biophysical Chemistry, Göttingen, Germany; ^2^Institute of Clinical Chemistry, University Medical Center Göttingen, Göttingen, Germany; ^3^Bioanalytical Mass Spectrometry Group, Max Planck Institute for Biophysical Chemistry, Göttingen, Germany; ^4^Department of Neurology, University Medical Center Göttingen, University of Göttingen, Göttingen, Germany; ^5^Cluster of Excellence Nanoscale Microscopy and Molecular Physiology of the Brain, Göttingen, Germany; ^6^Center for Biostructural Imaging of Neurodegeneration, University Medical Center Göttingen, Göttingen, Germany; ^7^NMR-Based Structural Biology Department, Max Planck Institute for Biophysical Chemistry, Göttingen, Germany; ^8^German Center for Neurodegenerative Diseases, DZNE, Göttingen, Germany; ^9^Cluster of Excellence “Multiscale Bioimaging: From Molecular Machines to Networks of Excitable Cells” (MBExC), University of Göttingen, Göttingen, Germany

**Keywords:** α-Synuclein, Parkinson’s disease, transgenic mouse, truncation, oligomerization, motor impairment, neuroinflammation, olfaction deficiency

## Abstract

α-synuclein (αSyn) is the main protein component of Lewy bodies, intracellular inclusions found in the brain of Parkinson’s disease (PD) patients. Neurotoxic αSyn species are broadly modified post-translationally and, in patients with genetic forms of PD, carry genetically encoded amino acid substitutions. Mutations and C-terminal truncation can increase αSyn oligomerization and fibrillization. Although several genetic mouse models based on αSyn mutations and/or truncations exist, there is still a lack of mouse models for synucleinopathies not relying on overexpression. We report here two synucleinopathy mouse models, which are based on a triple alanine to proline mutation and a C-terminal truncation of αSyn, but do not overexpress the mutant protein when compared to the endogenous mouse protein. We knocked h*αSyn^*TP*^* or h*αSyn^Δ119^* (h stands for “human”) into the murine *αSyn* locus. hαSyn^TP^ is a structure-based mutant with triple alanine to proline substitutions that favors oligomers, is neurotoxic and evokes PD-like symptoms in *Drosophila melanogaster*. hαSyn^Δ119^ lacks 21 amino acids at the C-terminus, favors fibrillary aggregates and occurs in PD. Knocking-in of h*αSyn^*TP*^* or h*αSyn^Δ119^* into the murine *αSyn* locus places the mutant protein under the control of the endogenous regulatory elements while simultaneously disrupting the *mαSyn* gene. Mass spectrometry revealed that h*αSyn^*TP*^* and h*αSyn^Δ119^* mice produced 12 and 10 times less mutant protein, compared to mαSyn in wild type mice. We show phenotypes in 1 and 1.5 years old *hαSyn^*TP*^* and *hαSyn^Δ119^* mice, despite the lower levels of hαSyn^TP^ and hαSyn^Δ119^ expression. Direct comparison of the two mouse models revealed many commonalities but also aspects unique to each model. Commonalities included strong immunoactive state, impaired olfaction and motor coordination deficits. Neither model showed DAergic neuronal loss. Impaired climbing abilities at 1 year of age and a deviant gait pattern at 1.5 years old were specific for *hαSyn^Δ119^* mice, while a compulsive behavior was exclusively detected in *hαSyn^*TP*^* mice starting at 1 year of age. We conclude that even at very moderate levels of expression the two αSyn variants evoke measurable and progressive deficiencies in mutant mice. The two transgenic mouse models can thus be suitable to study αSyn-variant-based pathology *in vivo* and test new therapeutic approaches.

## Introduction

Alpha-synuclein (αSyn) is a pre-synaptic 140 amino acid long, intrinsically disordered protein (Spillantini et al., 1997; Bendor et al., 2013). αSyn is expressed ubiquitously at high levels in the brain (Lein et al., 2007; Sjöstedt et al., 2020) and it is thought to play a role in synaptic maintenance by modulating synaptic vesicle recycling (Lotharius and Brundin, 2002) and vesicle fusion (Burre et al., 2010). αSyn is the main protein component of Lewy bodies, intracellular inclusions found in the brain of Parkinson’s disease (PD) patients (Spillantini et al., 1997). Aggregation of αSyn has been directly linked to PD and point mutations within the *αSyn* gene cause an early-onset, familial form of PD (Polymeropoulos et al., 1997; Kruger et al., 1998; Bendor et al., 2013). Furthermore, multiple copies of the *αSyn* gene evoke early-onset familial PD; the age of onset and disease severity depends on gene copy number and hence on αSyn dosage (Singleton et al., 2003; Farrer et al., 2004; Ross et al., 2008; Guhathakurta et al., 2017).

Parkinson’s disease primarily affects the nigro-striatal dopaminergic system (Hindle, 2010). Lewy body inclusions are the histological hallmark of PD, while the clinical phenotype includes tremor, rigidity, and bradykinesia (Jankovic, 2008; Meade et al., 2019). Other non-motor symptoms such as olfactory deficits, constipation, sleep disorder and impulsivity are also present and can precede motor defects by decades, although these non-motor symptoms originate beyond the basal ganglia (Smith et al., 2012). Motor symptoms are apparent only after >50% of DAergic neurons have been lost and pathology in the brain is by then advanced (Bobela et al., 2014).

Many current animal models involve human αSyn (hαSyn) or its genetic variants expressed under strong promoters in the brain of rodents (Bobela et al., 2014; Konnova and Swanberg, 2018). This up to 30-fold overexpression results in a range of neuropathological and behavioral phenotypes (Fleming et al., 2005). Pathology in these models appears as early as 2 months of age and can lead to paralysis and early death, as seen in the case of the A53T mutation (Giasson et al., 2002). Many overexpression-based models display cardinal features of PD; however, few models are currently available of early PD disturbances (Smith et al., 2012). A well-characterized model recapitulating important aspects of PD progression is the MI2 mouse in which a truncated hαSyn, hαSyn1–120, is expressed under the control of the tyrosine hydroxylase (TH) promoter in a mouse lacking endogenous αSyn (Wegrzynowicz et al., 2019). Another transgenic model utilizing the TH promoter conditionally expresses αSyn119 in a Cre-dependent manner (Daher et al., 2009).

Transgenic humanized models have been crucial for research on neurodegeneration (Nair et al., 2019). “Physiological” models aim to maintain expression of the targeted gene at endogenous levels in the appropriate cellular and temporal context (Nair et al., 2019). Physiological models have been made to recreate the unique human protein biochemistry (Nair et al., 2019) and to achieve the greatest physiological relevance in disease modeling or therapeutic development (Zhu et al., 2019). Endogenous expression levels are particularly important when modeling dosage-sensitive genes (Zhu et al., 2019). Typically, knock-in models targeting a “safe harbor locus” (e.g., Rosa26) or the gene of interest are created when attempting to generate physiological models (Nair et al., 2019). Although these gene-targeted mice tend to have slower progressing and milder phenotypes than overexpression-based models, they avoid overexpression artifacts, ectopic expression and mutations resulting from the random insertion of the transgenes (Zhu et al., 2019). This more subtle genetic manipulation may still nonetheless affect protein expression, and even the phenotype, as non-coding sequences (such as promoters) may be different in mouse and human (Nair et al., 2019). Features providing spatio-temporal control of expression (e.g., loxP sites) requires the insertion of additional exogenous sequences, which may compromise a faithful endogenous expression level or have other unintended effects (Zhu et al., 2019).

Here we developed two mouse models of synucleinopathies. Thus, we knocked-in variants of hαSyn into the endogenous mouse *αSyn* locus (*mαSyn*) bringing the mutations under the control of the endogenous *mαSyn* regulatory regions. We generated an *hαSyn^Δ119^* mouse expressing hαSyn ^Δ119^, i.e., hαSyn with a C-terminal deletion, thus ending at amino acid 119. C-terminal truncations of hαSyn have higher aggregation propensity (Crowther et al., 1998) and truncated hαSyn has been found in human synucleinopathies (Baba et al., 1998). As very recently confirmed, hαSyn^Δ119^ is one of the most common forms of truncated hαSyn (Sorrentino and Giasson, 2020). The second mouse model generated carries an *hαSyn* gene variant with three alanine to proline substitutions at residues 30, 56, and 76, henceforth called *hαSyn^*TP*^*. We chose to generate this model because hαSyn^TP^ is known to favor an oligomeric conformation and does not aggregate into amyloid fibrils *in vivo* since β-strand conformation is impaired (Karpinar et al., 2009; Zweckstetter et al., 2010; Taschenberger et al., 2012). In addition, hαSyn^TP^ is more toxic than wild type (WT) hαSyn when expressed in HEK293T cells, primary midbrain neurons, *C. elegans* and *Drosophila* brains (Karpinar et al., 2009) and causes PD-like non-motor symptoms in *Drosophila* (Gajula Balija et al., 2011). Because the hαSyn^Δ119^ and hαSyn^TP^ variants are at opposite ends of the oligomerization/fibrillization spectrum, *hαSyn^*TP*^* and *hαSyn^Δ119^* mice might help to understand the pathophysiological consequences that these mutants have when present in non-overexpressing mammalian models. Additionally, the two models, which only differ in the *hαSyn* variant, allow for a direct comparison.

We show histological, biochemical and behavioral commonalities and differences in the *hαSyn^*TP*^* and *hαSyn^Δ119^* mice. The observed changes occur despite a 10 to 12-fold lower expression level of the hαSyn mutant proteins when compared to the mouse WT protein. Our data further suggest that the *hαSyn^*TP*^* and *hαSyn^Δ119^* mouse models can be suitable to assess therapeutic treatments, especially those aimed at early stages of PD.

## Results

### Generation of the *hαSyn^*TP*^* Mouse Line

**FIGURE 1:**
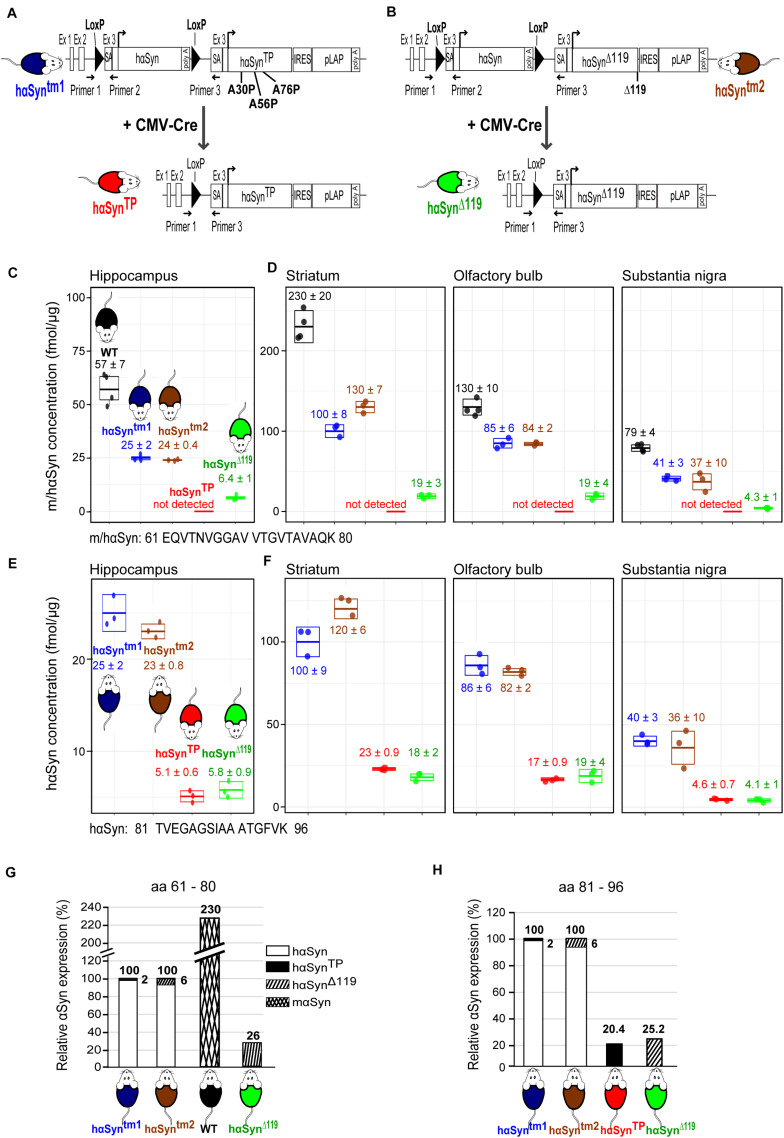
Generation of the *hαSyn^*TP*^* and *hαSyn^Δ119^* conditional mouse models and validation of αSyn expression using mass spectrometry. **(A)** To generate the conditional *hαSyn^*TP*^* mouse line, we knocked into the endogenous *mαSyn* locus a floxed-*hαSyn* cDNA followed by *hαSyn^*TP*^* (encoding A30P, A56P, and A76P substitutions) and a reporter gene. Additional regulatory sequences were also included. This generated the *hαSyn^*tm1*^* mice (targeted mutation 1, blue mouse top diagram). Upon Cre-recombination, *hαSyn^*TP*^* mice were generated (red mouse, bottom diagram). SA, splicing acceptor; IRES, internal ribosomal entry site; pLAP, placental alkaline phosphatase; and Primer 1, Primer 2, Primer 3 are genotyping primers. **(B)** We generated the *hαSyn^Δ119^* mouse following the same approach as in A except that *hαSyn^Δ119^* mice expressed a truncated *hαSyn* at position 119 (green mouse, bottom diagram). **(C)** MS analysis of hippocampal protein extracts from 1.5 years old mice detecting a peptide (aa 61–80) shared between mouse and human αSyn. The concentration of αSyn peptide (fmol/μg extracted protein) on the ordinate was calculated using ^15^N-labeled recombinant hαSyn spiked-in to tissue lysates. This peptide was not detected in *hαSyn^*TP*^* mice, indicating Cre-mediated excision of the *hαSyn* gene was complete. Values represent mean ± SD. WT, wild type. **(D)** MS analysis of protein extracts from the striatum, olfactory bulbs and substantia nigra of 1.5 years old mice detecting a peptide (aa 61–80) shared between mouse and human αSyn. This peptide was not detected in hαSynTP mice, indicating Cre-mediated excision of the hαSyn gene was complete. Values represent mean ± SD. WT, wild type. **(E)** MS analysis of hippocampal protein extracts from 1.5 years old mice detecting a pan peptide (aa 81–96) present in all variants of hαSyn but not in mαSyn. Values represent mean ± SD. **(F)** MS analysis of protein extracts from the striatum, olfactory bulbs and substantia nigra of 1.5 years old mice detecting a pan peptide (aa 81–96) present in all variants of hαSyn but not in mαSyn. Values represent mean ± SD. **(G)** *hαSyn^*tm1*^* and *hαSyn^*tm2*^* mice express 2.3 times less αSyn than WT mice. Note the small amount of hαSyn^TP^ and hαSyn^Δ119^ due to read-through transcription. Compared to WT, *hαSyn^*TP*^* mice express 11.2% αSyn. **(H)** *hαSyn^*TP*^* and *hαSyn^Δ119^* mice produced the desired mutant proteins; however, expression was approximately 20–25% of the hαSyn seen in the *hαSyn^*tm1*^* and *hαSyn^*tm2*^* strains.

Our first goal was to generate the hαSyn^TP^ mouse (Figure 1A, red mouse, lower diagram) as a conditional knock-in mouse line. Three alanine to proline substitutions at residues 30, 56, and 76 were incorporated in the hαSyn protein. To achieve our goal, we first constructed a targeting vector that was introduced into exon 3 of mouse *αSyn*, thereby disrupting the murine *αSyn* gene and placing the transgene under the control of the endogenous mouse regulatory region (Supplementary Figures 1A,B; section “Materials and Methods”). In essence, the targeting vector consisted of loxP flanked-*hαSyn*, followed by the mutant *hαSyn^*TP*^* and a *pLAP* reporter gene. Using embryonic stem cell technology, we generated the *hαSyn^*tm1*^* mouse strain (tm stands for targeted mutation) that contained the loxP flanked-*hαSyn*, followed by *hαSyn^*TP*^* and the *pLAP* reporter (Figure 1A, blue mouse, upper diagram). *hαSyn^*tm1*^* mice were crossed with CMV-Cre mice, which express Cre recombinase ubiquitously (Schwenk et al., 1995). This crossing should yield offspring lacking hαSyn protein but expressing hαSyn^TP^ instead (Figure 1A, red mouse). To demonstrate the absence of *hαSyn*, we used RT-PCR (for primers see section “Materials and Methods”) to detect a 512 bp amplicon that covered this gene. As expected, the amplicon was seen in the *hαSyn^*tm1*^* strain but was absent in *hαSyn^*TP*^* mice (Supplementary Figure 4A). Thus, Cre excision was complete. We corroborated the absence of hαSyn in *hαSyn^*TP*^* mice also by mass spectrometry (MS) using single reaction monitoring from hippocampal protein extracts. A peptide common to mouse and hαSyn (61 – EQVTNVGGV VTGVTAVAQK – 80) but not present in hαSyn^*TP*^, was detected in *hαSyn^*tm1*^* mice but not found in *hαSyn^*TP*^* mice (Figure 1C, blue and red mice), again indicating that Cre-mediated excision was complete.

Because the endogenous mouse sequence was not deleted but interrupted and exons 3 to 7 pushed downstream (Supplementary Figure 1A), we corroborated by MS that no peptide was generated from these exons 3 to 7. We did not detect the mouse-specific peptides 46 – EGVVHGVTTVAEK – 58 and 81 – TVEGAGNIAAATGFVK – 96 in any of the transgenic lines (Supplementary Figures 2, 3). These peptides are encoded within exons 4 and 5 and are located in the N-terminus and the NAC domain. In WT, both of these peptides generated strong signals (Supplementary Figures 2, 3).

We next examined whether the *hαSyn^*TP*^* gene gives rise to transcripts in *hαSyn^*TP*^* mice. A distinct 566 bp amplicon that included the *hαSyn^*TP*^* transcript was detected in cortical RNA of *hαSyn^*TP*^* mice (Supplementary Figure 4B, red). Of note, we detected this amplicon also in the cortex of *hαSyn^*tm1*^* mice, indicating read-through transcription in this tissue (Supplementary Figure 4B, blue). We corroborated this finding by MS. An hαSyn^*TP*^- specific peptide (61 – EQVTNVGGAV VTGVTPVAQK – 80) which should not be produced in *hαSyn^*tm1*^* mice was nonetheless present but at a 10-fold lower amount than in the *hαSyn^*TP*^* mice (Supplementary Figure 4C, blue vs. red mice).

Given that we attempted not to overexpress hαSyn^*TP*^, we compared the levels of *hαSyn* and *hαSyn^*TP*^* transcripts in *hαSyn^*tm1*^* and *hαSyn^*TP*^* mice. We used qPCR and pan primers that amplified both *hαSyn* and *hαSyn^*TP*^*. Since *hαSyn^*TP*^* mice lack *hαSyn* (Supplementary Figure 4A) the amplicon exclusively represented *hαSyn^*TP*^* mRNA. qPCR analysis showed that there was 13 times higher cortical expression of *hαSyn* transcripts in *hαSyn^*tm1*^* than mutant transcript in *hαSyn^*TP*^* mice (Supplementary Figure 4D, left bar). This indicated mutant transcript was not overexpressed but in fact present at a substantially lower level than WT transcript. A reduction was also seen for hαSyn^TP^ protein (section “hαSyn^TP^ and hαSyn^Δ^ ^119^ proteins are expressed in the hippocampus, striatum, substantia nigra (SN) and olfactory bulb”).

### Generation of the *hαSyn^Δ119^* Mouse Line

We next generated the *hαSyn^Δ119^* mouse model in which a C-terminally truncated hαSyn was expressed (Figure 1B, green mouse, lower diagram) applying the same strategy as described above. The corresponding targeting vector is shown in Supplementary Figure 1B. The generated mice were termed *hαSyn^*tm2*^* (Figure 1B, brown mouse). We mated *hαSyn^*tm2*^* to CMV-Cre mice leading to *hαSyn^Δ119^* mice (Figure 1B, green mouse). We found complete Cre-mediated excision of floxed-h*αSyn* in *hαSyn^Δ119^* mice, as we did not detect the 512 bp *hαSyn* amplicon in mRNA from *hαSyn^Δ119^* mice (Supplementary Figure 4A, green). Next, we assayed for *hαSyn^Δ119^* transcripts in *hαSyn^Δ119^* mice. We detected by RT-PCR a distinct 503 bp amplicon covering *hαSyn^Δ119^* in mutant mice (Supplementary Figure 4B, green). Subsequent qPCR of cortical cDNA showed that mutant mRNA in *hαSyn^Δ119^* mice was 9 times less abundant than the *hαSyn* mRNA in *hαSyn^*tm2*^* strain (Supplementary Figure 4D, right bar). MS analyses of hippocampal protein extracts detected an hαSyn^Δ119^-specific peptide (102 – KNEEGAPQE GILEDMPVD – 119) in the *hαSyn^*tm2*^* mice but at an approximately 4-fold lower level than in *hαSyn^Δ119^* mice (Supplementary Figure 4E, brown vs. green mouse). The presence of hαSyn^Δ119^ protein in *hαSyn^*tm2*^* mice is likely due to read-through transcription.

### hαSyn^TP^ and hαSyn^Δ119^ Proteins Are Expressed in the Hippocampus, Striatum, Substantia Nigra and Olfactory Bulb

The significantly reduced levels of mutant *hαSyn* mRNA (Supplementary Figure 4D) translates into a similar reduction of mutant protein. We used MS to assess the concentration of αSyn in hippocampus, striatum, SN and olfactory bulb. We first compared the levels of murine αSyn protein in a WT hippocampus with hαSyn protein in hippocampus of the *hαSyn^*tm1*^* mouse. A peptide common to mouse and hαSyn (61 – EQVTNVGGAV VTGVTAVAQK – 80), was reduced by a factor of 2.3 in *hαSyn^*tm1*^* mice (57 ± 7 fmol/μg total protein vs. 25 ± 2 fmol/μg, Figure 1C, black vs. blue). This indicates that the mαSyn protein in the hippocampus of a WT mouse was expressed at a 2.3-times higher level than hαSyn in the *hαSyn^*tm1*^* knock-in model. A similar fold-reduction of expression was observed in the other brain regions, although the absolute αSyn protein amount varied between the different regions (Figure 1D).

Next, we compared the levels of hαSyn and hαSyn^TP^ in the *hαSyn^*tm1*^* and *hαSyn^*TP*^* mice. We measured the levels of a pan peptide 81 – TVEGAGSIAAATGFVK – 96, present in all variants of hαSyn. In the hippocampus, we found a 5-fold decrease for this peptide in *hαSyn^*TP*^* compared to *hαSyn^*tm1*^* mice (25 ± 2 fmol/μg vs. 5.1 ± 0.6 fmol/μg, Figure 1E, blue vs. red). In other words, the hαSyn^TP^ protein was about 5 times less abundant in *hαSyn^*TP*^* mice than the hαSyn protein in *hαSyn^*tm1*^* mice. Relative to mαSyn in WT mice, hαSyn^TP^ was 12 times less abundant (2.3 times 5). A similar decrease for the 81 – TVEGAGSIAA ATGFVK – 96 peptide was also seen in the other brain regions examined (Figure 1F). Ideally, the stepwise quantification between WT, *hαSyn^*tm1*^* and *hαSyn^*TP*^* mice should be pursued with a pan-peptide. Such a peptide was not available with the tryptic digests. However, we feel confident with our quantification since both 61 – EQVTNVGGAVVTGVTAVAQK – 80 and 81 – TVEGAGSIAAATGFVK – 96 peptides yielded numerically very similar results (hippocampus Figures 1C,E; other brain regions Figures 1D,F). Quantification of the different *α*Syn species by Western blotting turned out to be problematic. E.g., densitometry did not show the reduction of hαSyn^TP^ protein that MS had detected. Presumably, the presence of the mutation affected antibody affinity.

The mouse model harboring the C-terminal truncation also showed a reduced mutant αSyn expression. In the hippocampus, we found 2.4-times less 61 – EQVTNVGGAVVTGVTAVAQK – 80 peptide in transgenic *hαSyn^*tm2*^* mice relative to WT (57 ± 7 fmol/μg vs. 24 ± 0.4 fmol/μg, Figure 1C, black vs. brown). Comparison of *hαSyn^*tm2*^* to *hαSyn^Δ119^* mice revealed a further 4-fold decrease in peptide amount in *hαSyn^Δ119^* mice (24 ± 0.4 fmol/μg vs. 6.4 ± 1 fmol/μg, Figure 1C, brown vs. green). Direct comparison of WT to *hαSyn^Δ119^* mice showed an approx. 10-fold decrease in peptide amount (57 ± 7 fmol/μg vs. 6.4 ± 1 fmol/μg, Figure 1C, black vs. green). A decrease by a factor of 10 was also seen in striatum, SN and olfactory bulb (Figure 1D). Comparing *hαSyn^*tm2*^* and *hαSyn^Δ119^* mice, we found that the 81 – TVEGAGSIAAATGFVK – 96 peptide was decreased 4-fold in all brain regions (hippocampus: Figure 1E; other brain regions Figure 1F). Thus, both peptides yielded the same results.

This work has generated four mouse lines that differ in their content of human WT and mutant αSyn. Figures 1G,H summarize the relative expression levels of the hαSyn variants in the four lines. In *hαSyn^*tm1*^* mice, the ratio of hαSyn to hαSyn^TP^ is 98 to 2. The small fraction of hαSyn^TP^ is due to read-through transcription. In *hαSyn^*tm2*^* mice, the ratio of hαSyn to hαSyn^Δ119^ is 94 to 6 (Figure 1G). Thus, these two genotypes are predominantly making hαSyn. The level of expression of hαSyn in the *hαSyn^*tm1*^* and *hαSyn^*tm2*^* lines is~ 2.3 times lower than that of mαSyn in a WT mouse (Figure 1G). When one crosses *hαSyn^*tm1*^* and *hαSyn^*tm2*^* parents with a Cre-driver, the *hαSyn* gene is completely excised. The resulting offspring *hαSyn^*TP*^* and *hαSyn^Δ119^* mice produce the desired mutant proteins. However, expression amounts only up to approx. a quarter of the hαSyn seen in the *hαSyn^*tm1*^* and *hαSyn^*tm2*^* strains (Figure 1H) and only~ 11% of the mαSyn expressed in WT mice. Thus, none of the mouse models overexpresses the *hαSyn^*TP*^* or *hαSyn^Δ119^* transgenes.

### Neuroinflammatory Response in 1.5 Years Old *hαSyn^*TP*^* and *hαSyn^Δ119^* Mice

**FIGURE 2:**
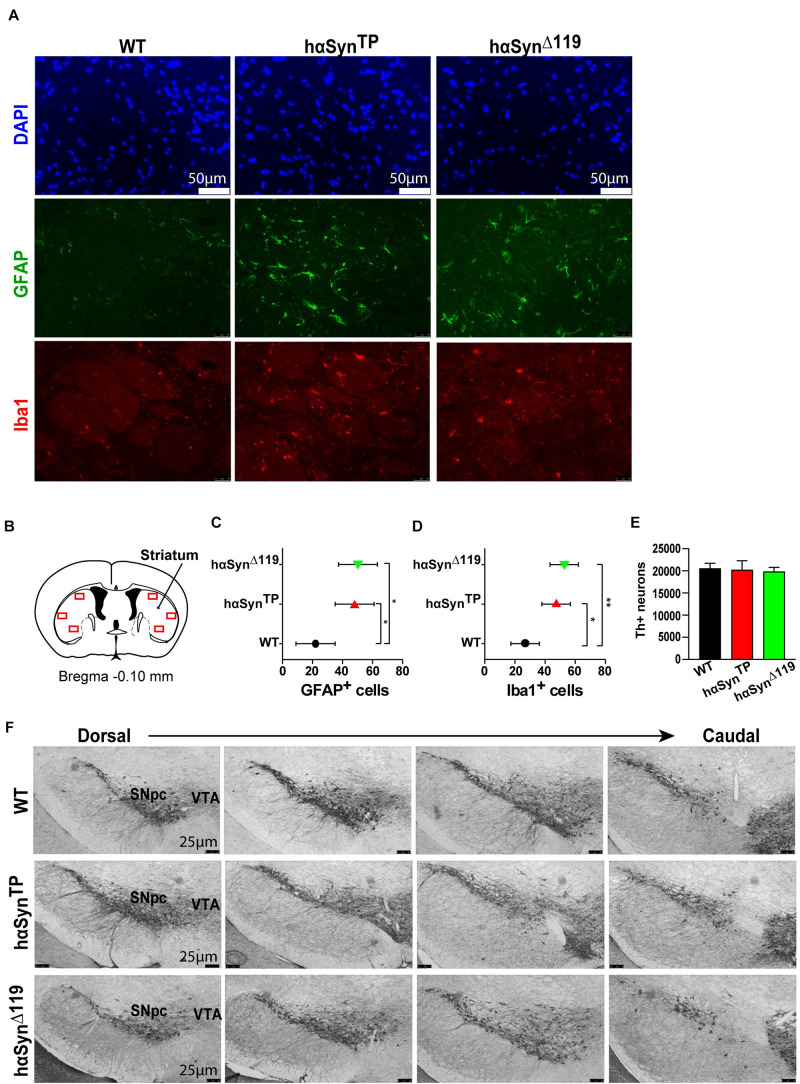
Active neuroinflammatory response in 1.5 years old *hαSyn^*TP*^* and *hαSyn^Δ119^* mice but no apparent loss of DAergic neurons of the substantia nigra. **(A)** Representative coronal sections through the striatum from perfused WT, *hαSyn^*TP*^* and *hαSyn^Δ119^* mice immunostained with GFAP (green) and Iba1 (red) showed more GFAP^+^ and Iba1^+^ cells in *hαSyn^*TP*^* and *hαSyn^Δ119^* mice. DAPI shows nuclei in blue. **(B)** Striatal regions (red rectangles, 3 regions per hemisphere) selected for quantification of GFAP^+^ and Iba1^+^ cells. Image modified from the Mouse Brain Atlas (Franklin and Paxinos, 2013). **(C)** Quantification of GFAP^+^ cells in striatal coronal sections showed a significant increase of reactive astrocytes in *hαSyn^*TP*^* and *hαSyn^Δ119^* mice compared to WT mice. Represented in graph are group means ± SEM. Group comparisons using Nested 1-way ANOVA with Tukey’s multiple comparisons test. *p < 0.05, **p < 0.01. **(D)** Quantification of Iba1^+^ cells in striatal coronal section showed a significant increase of reactive microglia in *hαSyn^*TP*^* and *hαSyn^Δ119^* mice compared to WT mice. Analyses as in **C**. **(E)** Stereological quantification of Tyrosine hydroxylase (Th) positive neurons in the substantia nigra of 1.5 years old mice showed a similar number of positive DAergic neurons for all groups, suggesting no neuronal loss occurs in the *hαSyn^*TP*^* or *hαSyn^Δ119^* models. *n* = 3 per group. Comparison to WT using 1-way ANOVA with Dunnett’s multiple comparisons test. **(F)** Representative coronal brain sections from perfused WT, *hαSyn^*TP*^* and *hαSyn^Δ119^* mice immunostained with anti-Tyrosine hydroxylase antibody, a marker for DAergic neurons in the substantia nigra pars compacta (SNpc) and ventral tegmental area (VTA). There were no apparent morphological changes in *hαSyn^*TP*^* or *hαSyn^Δ119^* compared to WT mice.

For the cell-biological and behavioral analyses, we compared WT, *hαSyn^*TP*^* and *hαSyn^Δ119^* 1.5 year-old male mice. It has been well documented that neuroinflammation characterizes neurodegenerative diseases, including PD (Qian et al., 2010; Doty et al., 2015; Pajares et al., 2020). Thus, we assessed neuroinflammation in our models. We probed for astrocyte and microglia activation, using GFAP and Iba1 antibodies, on coronal brain sections from perfused mice (Figure 2A and Supplementary Table 1). We included three mice per group, four striatal sections per brain, three striatal regions per hemisphere (Figure 2B) and counted the number of GFAP^+^ and Iba1^+^ cells.

Wild type mice showed a low number of GFAP^+^ cells, while *hαSyn^*TP*^* and *hαSyn^Δ119^* mice showed at least twice as many positive cells (Figure 2C and Supplementary Table 1), indicating a strong activation of astrocytes in the striatum of mutants. Congruently, we found the lowest number of Iba1^+^ cells in WT mice and twice as many cells were positive in *hαSyn^*TP*^* and *hαSyn^Δ119^* mice (Figure 2D and Supplementary Table 1). The doubling of Iba1^+^ cells indicated a strong microglia activation in mutant mice compared to WT. We also profiled gene expression of several cytokines by qPCR in striatal samples. We detected a trend for upregulation of these genes in both *hαSyn^*TP*^* and *hαSyn^Δ119^* mice. Notably, the chemokines *KC/Gro*, *MCP-1*, *MIP-1α* and *MIP-1β* were significantly up-regulated but exclusively in *hαSyn^Δ119^* mice (Supplementary Figure 5).

### No Neuronal Loss in *hαSyn^*TP*^* and *hαSyn^Δ119^* Mice

Many synucleinopathies, including PD, are associated with DAergic neuronal loss (Savitt et al., 2006). Thus, to investigate whether neuroinflammation or behavioral alterations (see below) in the *hαSyn^*TP*^* and *hαSyn^Δ119^* mouse models were associated with DAergic neuronal loss, we immunostained for TH, a marker of DAergic neurons. Coronal brain sections encompassing the SN in *hαSyn^*TP*^*, *hαSyn^Δ119^* and WT mice at 1.5 years of age were stained with anti-Th antibody. Stereological quantification of Th+ neurons showed a similar number of DAergic nigral neurons for all groups, suggesting that no neuronal loss occurred (Figure 2E). A visual comparison of Th immunoreactivity revealed comparable patterns in all genotypes throughout the region (Figure 2F). The gross size and shape of the SN and the ventral tegmental area (VTA) appeared to be similar, indicating the absence of visible morphological changes.

### Increased Variability of the Concentration of Dopamine and Its Metabolites in the Striatum of *hαSyn^*TP*^* and *hαSyn^Δ119^* Mice

**FIGURE 3:**
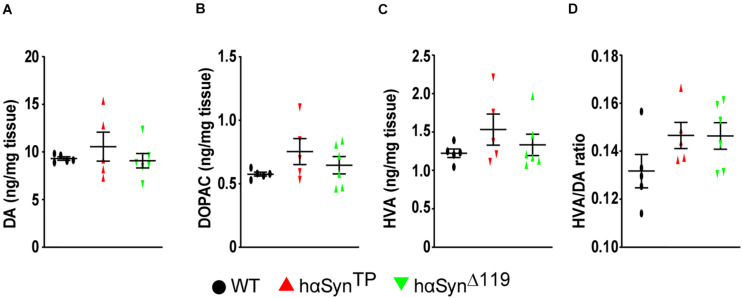
Increased variability of the concentration of dopamine and its metabolites in the striatum of *hαSyn^*TP*^* and *hαSyn^Δ119^* mice at 1.5 years of age. **(A–C)** DA, DOPAC, and HVA content varied considerably within *hαSyn^*TP*^* or *hαSyn^Δ119^*, but not within WT mice. **(D)** *hαSyn^*TP*^* and *hαSyn^Δ119^* mice showed increased HVA/DA ratio compared to WT, although not statistically significant. For all instances, *n* ≥ 5 mice per group. Represented in graph are individual values and group means ± SEM. Group comparisons using 1-way ANOVA with Bonferroni’s multiple comparisons test for mean differences and Bartlett’s test for SDs differences.

We quantified dopamine (DA) and its metabolites, 3,4-dihydroxyphenylacetic acid (DOPAC) and homovanillic acid (HVA), in the striatum of 1.5 year-old mice by high performance liquid chromatography (Figures 3A–C). In general, there was no significant difference between groups (Figure 3 and Supplementary Table 1). We found that metabolite amounts were very similar among individuals within the WT group, but varied considerably within the *hαSyn^*TP*^* and *hαSyn^Δ119^* groups (Figures 3A–C and Supplementary Table 1). We also found that the HVA/DA ratio was similar in *hαSyn^*TP*^* and *hαSyn^Δ119^* mice and potential differences to WT did not reach statistical significance (Figure 3D and Supplementary Table 1).

### Motor and Non-Motor Impairment in *hαSyn^*TP*^* and *hαSyn^Δ119^* Mice

To establish whether the expression of hαSyn^TP^ and hαSyn^Δ119^ affected motor and non-motor abilities, a cohort of male mice underwent a battery of motor and behavioral tests at 1 and 1.5 years of age. Motor function was investigated by the vertical pole, rotarod and CatWalk^®^ gait tests. Non-motor behavior was examined using the buried food olfactory test and the nestlet-shredding test.

#### Vertical Pole Climbing Impairment in *hαSyn^*TP*^* and *hαSyn^Δ119^* Mice

**FIGURE 4:**
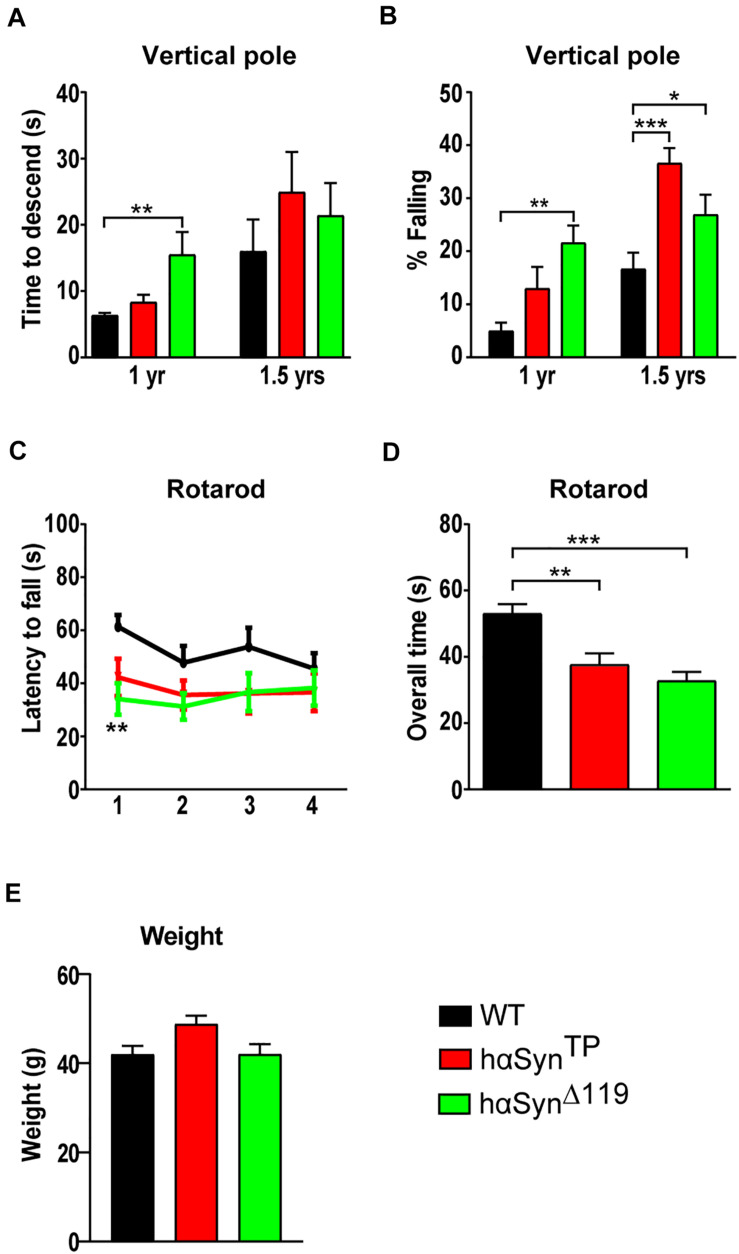
Progressive and age-dependent motor impairment in *hαSyn^*TP*^* and *hαSyn^Δ119^* mice at 1 and 1.5 years of age. **(A,B)** Vertical pole test at 1 and 1.5 years of age. **(A)** shows the time of decent and **(B)** the percentage of mice falling. Group comparisons using 1 way ANOVA with Tukey’s multiple comparisons test **(A)** or 2-way ANOVA with Bonferroni’s multiple comparisons test **(B)**. **(C)** *hαSyn^*TP*^* and *hαSyn^Δ119^* mice consistently had a shorter latency to fall off the accelerating rotarod compared to WT mice over all four trials. Group comparisons using 2-way ANOVA with Bonferroni’s multiple comparisons test. **(D)** *hαSyn^*TP*^* and *hαSyn^Δ119^* mice spent overall significantly less time on the accelerating rotarod than WT mice at 1.5 years of age. Group comparisons using 1-way ANOVA with Tukey’s multiple comparisons test. **(E)** Weight of 1.5-year-old WT, *hαSyn^*TP*^* and *hαSyn^Δ119^* mice. Group comparisons using 1-way ANOVA with Tukey’s multiple comparisons test. For all experiments, values represent mean ± SEM. *p < 0.05, **p < 0.01, and ***p < 0.001.

The vertical pole test (Ogawa et al., 1985; Matsuura et al., 1997) is well-established for examining bradykinesia and is regarded as highly sensitive for nigrostriatal dysfunction (Hölter and Glasl, 2012). We quantified the time it took mice to turn around and descend the pole. In case mice fell, a max score of 60 s was given. At both ages, there was no significant difference in the performance between the genotypes except for *hαSyn^Δ119^* mice, which descended slower at the 1-year time point (Figure 4A and Supplementary Table 2). Then, we compared the percentage of mice falling. At 1 year of age, *hαSyn^Δ119^* mice stood out; the percentage of mutant mice falling was four times greater than for WT (Figure 4B and Supplementary Table 2). 1-year old *hαSyn^*TP*^* mice had a tendency to fall more often than WT mice, although this difference was not significant. However, by 1.5 years of age, *hαSyn^*TP*^* mice fell most frequently and surpassed *hαSyn^Δ119^* mice (Figure 4B and Supplementary Table 2).

#### Accelerating Rotarod Motor Impairment in *hαSyn^*TP*^* and *hαSyn^Δ119^* Mice

We subjected 1.5 year-old mice in the accelerating rotarod test (Hölter and Glasl, 2012). We trained mice over 2 days to walk on a rod rotating at a constant speed. On days 3 and 4, we tested mice on a gradually accelerating rod (5 to 40 rpm). Mice underwent trials twice a day with a 5-h inter-trial rest period. We recorded latency to fall off the rod (Figures 4C,D and Supplementary Table 3). Overall, the performance of *hαSyn^*TP*^* and *hαSyn^Δ119^* mice clearly separated from that of WT mice (Figure 4C). Individuals expressing mutant protein fell off the rod consistently faster over all trials, although only trial one reached significance for *hαSyn^Δ119^* mice. Comparison of the overall time spent on the rod revealed a strong and significant difference between the genotypes. Specifically, animals that expressed mutant hαSyn fell off the rod faster (Figure 4D and Supplementary Table 3). Because weight can affect performance of this test (McFadyen et al., 2003), we compared the weight in all groups of mice. Weight comparison revealed differences in weight, although no statistical significance was reached (Figure 4E). However, there was no correlation between rotarod performance and weight. WT and *hαSyn^Δ119^* mice were similar in weight but highly differed in rotarod performance.

#### Affected Gait Parameters in *hαSyn^*TP*^* and *hαSyn^Δ119^* Mice

**FIGURE 5:**
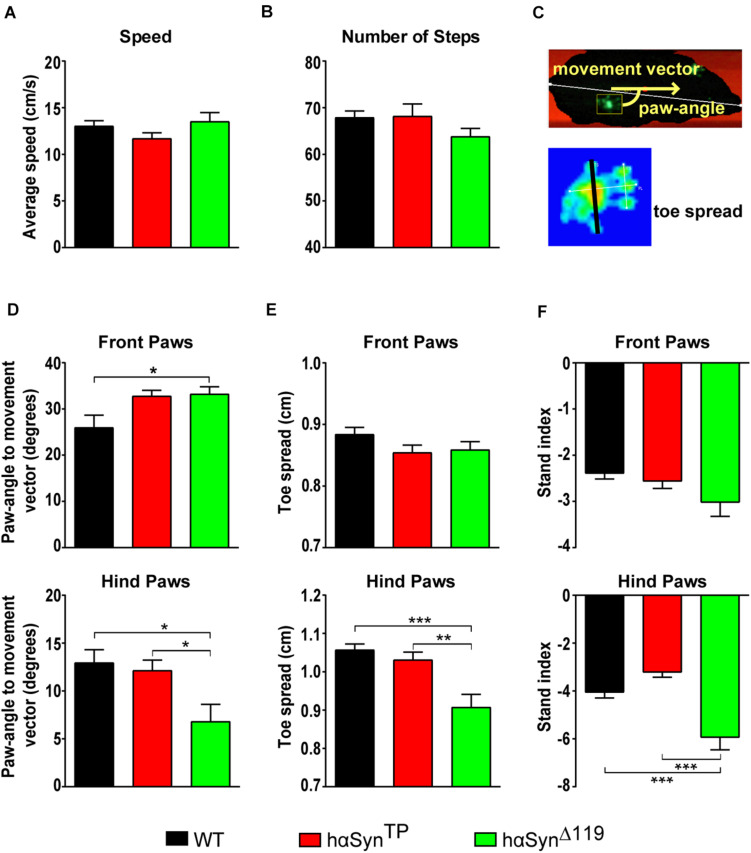
Deviant gait patterns in *hαSyn^*TP*^* and *hαSyn^Δ119^* mice at 1.5 years of age. **(A,B)** Gait analysis using the CatWalk system in WT, *hαSyn^*TP*^*, and *hαSyn^Δ119^* mice showed no significant difference in their walking speed **(A)** or the number of steps **(B)**. **(C)** Diagram illustrating the “paw-angle to movement vector” and the toe spread (black line between toes 1 and 5). **(D–F)** Comparison of paw-angle, toe spread and stand index for front (top graphs) and hind paws (bottom graphs) for the various genotypes. Values represent mean ± SEM. Group comparisons using 1-way ANOVA with Tukey’s multiple comparisons test. *p < 0.05, **p < 0.01, and ***p < 0.001.

We performed quantitative gait analysis of voluntary walking using the CatWalk^®^ gait analysis system (Tatenhorst et al., 2016). The CatWalk system consists of a linear walkway on a glass plate internally reflecting light except at those places where the foot makes contact with the plate. There light is refracted to the opposite side, illuminating the areas of contact. A high-speed camera, placed underneath, captures these contacts and the CatWalk XT gait analysis software automatically computes footprints, along with many other gait parameters. In our assays, 1.5-year old mice performed three runs and means out of these runs were compared. Notably, neither speed nor the number of steps were significantly different between *hαSyn^*TP*^* or *hαSyn^Δ119^* and WT mice (Figures 5A,B and Supplementary Table 4A). Three parameters, paw-angle to movement vector, toe spread (Figure 5C) and stand index were clearly affected in mutant mice. With regard to the paw-angle to movement vector, αSyn mutant models showed an increase for the front paws, although only significant for *hαSyn^Δ119^* mice (Figure 5D and Supplementary Table 4B). For the hind paws, *hαSyn^Δ119^* mice showed a significantly smaller paw angle relative to *hαSyn^*TP*^* and WT mice (Figure 5D and Supplementary Table 4B). The distance between the first and fifth toe (toe spread) was significantly smaller in the hind paws of *hαSyn^Δ119^* mice, compared to all other genotypes (Figure 5E and Supplementary Table 4B). The stand index was also affected. This index is a measure for the speed at which the paws lose contact with the glass plate. Stand index was higher for all four paws in *hαSyn^Δ119^* compared to WT mice, although only statistically significant for the hind paws (Figure 5F and Supplementary Table 4B). The hind paws of *hαSyn^*TP*^* mice showed a trend in the opposite direction (Figure 5F and Supplementary Table 4B). Overall, it appears that abnormalities in gait parameters are most pronounced in *hαSyn^Δ119^* mice.

#### Olfactory Impairment in *hαSyn^*TP*^* and *hαSyn^Δ119^* Mice

**FIGURE 6:**
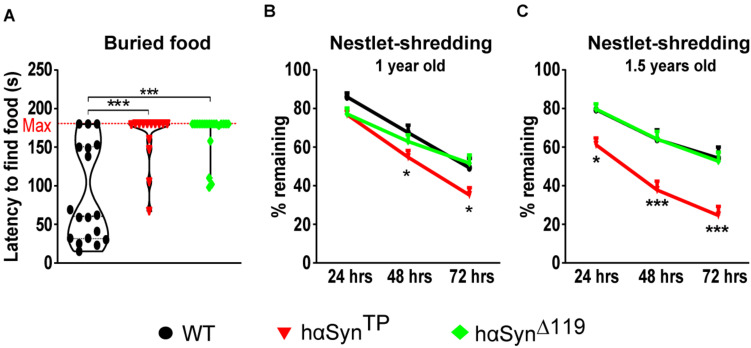
Olfactory impairment and abnormal nestlet-shredding behavior in *hαSyn^*TP*^* and *hαSyn^Δ119^* mice. **(A)** Buried food test showed 1.5 years old *hαSyn^*TP*^* and *hαSyn^Δ119^* mice were mostly unable to find the buried cereal ball. Group comparisons using 1-way ANOVA with Tukey’s multiple comparisons test. **(B,C)** Nestlet-shredding for 1 and 1.5 years old WT, *hαSyn^*TP*^*, and *hαSyn^Δ119^* mice was compared. Group comparisons using 2-way ANOVA with Bonferroni’s multiple comparisons test. Values in **(A–C)** represent mean ± SEM. *p < 0.05, and ***p < 0.001.

An early and preclinical sign of PD in humans is hyposmia, the decreased ability to detect odors (Ponsen et al., 2004; Mahlknecht et al., 2015). Thus, we assessed the sense of smell for all genotypes using the buried food test (Yang and Crawley, 2009; Hall et al., 2015). After two habituation days, we quantified the time it took mice to find a Cocoa Puff^®^ ball (Kellogg’s, Battle Creek, MI, United States) buried approx. 1 cm deep in the bedding material. We assigned the max score (180 s) when mice were unable to find the cereal ball within the duration of the experiment. We found a highly significant olfactory impairment in both *hαSyn^*TP*^* and *hαSyn^Δ119^* mice relative to WT mice (Figure 6A and Supplementary Table 5). Although WT segregated in good and bad performers (Figure 6A and Supplementary Table 5), 11 out of 18 consistently found the cocoa ball within 30–60 s. In the case of *hαSyn^*TP*^* mice, only 4 out of 14 mice were able to retrieve the cocoa ball after 2 min (Figure 6A and Supplementary Table 5). Similarly, only 4 out of 20 *hαSyn^Δ119^* mice were able to retrieve the cocoa ball during the last minute of the experiment (Figure 6A and Supplementary Table 5). These differences were highly significant (Figure 6A and Supplementary Table 5), indicating that the sense of smell in *hαSyn^*TP*^* and *hαSyn^Δ119^* mice was impaired.

#### Abnormal Nestlet-Shredding Behavior in *hαSyn^Δ119^* Mice

Another aspect of PD is the presence of compulsive behavior (Erga et al., 2017). To investigate whether mice expressing mutant αSyn protein show a repetitive and compulsive-like behavior, we performed the nestlet-shredding test (Witkin, 2008). We placed pre-weighed cotton nestlet pads onto the cage’s metallic grid lid and weighted the remaining material after 24, 48, and 72 h. We performed the test with mice at 1 and 1.5 years of age (Figures 6B,C and Supplementary Table 5). At 1 year of age, *hαSyn^*TP*^* mice consistently had less nesting material left compared to WT mice after 48 and 72 h (Figure 6B and Supplementary Table 5). By 1.5 years of age, *hαSyn^*TP*^* mice showed a clear and significantly different behavior compared to WT mice (Figure 6C and Supplementary Table 5). The remaining nesting material in cages from *hαSyn^*TP*^* mice decreased by 23, 41, and 55% after 24, 48, and 72 h, respectively. Notably, *hαSyn^Δ119^* mice performed indistinguishably from WT mice (Figures 6B,C and Supplementary Table 5).

## Discussion

We created mouse strains in which either *hαSyn^*TP*^* or *hαSyn^Δ119^* mutations were inserted into exon 3 of the murine *αSyn* gene. In these knock-in mice, the endogenous *αSyn* gene was thus disrupted and the expression of the transgenes was placed under the control of the murine *αSyn* regulatory region. These mice were generated in a two-step process. First, through recombineering and embryonic stem cell technology, we generated mice carrying a transgene inserted into exon 3 of mouse *αSyn*. In essence, this transgene consisted of the loxP flanked-*hαSyn* gene, followed by either *hαSyn^*TP*^* or *hαSyn^Δ119^* and a *pLAP* reporter. The resulting mouse strains were termed *hαSyn^*tm1*^* and *hαSyn^*tm2*^*. In a second step, *hαSyn^*tm1*^* and *hαSyn^*tm2*^*mice were crossed with CMV-Cre mice, which express Cre recombinase ubiquitously. This crossing should result in mice with a complete loss of hαSyn protein concomitant with the expression of hαSyn^TP^ or hαSyn^Δ119^.

In principle, *hαSyn^*tm1*^* and *hαSyn^*tm2*^* mice should express human WT and, upon Cre-recombination, mutant transgenes at a level similar to murine αSyn in a WT mouse. MS quantification of mαSyn and hαSyn proteins from several brain regions of WT, *hαSyn^*tm1*^* and *hαSyn^*tm2*^* mice showed that transgenic mice had approximately 2 times lower hαSyn protein than WT mice had mαSyn. We also noticed that in the hippocampus of *hαSyn^*tm1*^* and *hαSyn^*tm2*^* mice, 2 and 6% (by mass) of mutant protein was present. This indicates that the stop signal at the 3′ end of hαSyn was slightly leaky.

Crossing *hαSyn^*tm1*^* and *hαSyn^*tm2*^* mice with CMV-Cre mice resulted in the expected complete loss of WT hαSyn protein concomitant with expression of hαSyn^TP^ or hαSyn^Δ119^ proteins. Both of these proteins were detected by MS, albeit at a level about four-five times lower than the expression of hαSyn in the *hαSyn^*tm1*^* and *hαSyn^*tm2*^* strains. mRNA levels were also reduced, suggesting that less efficient transcription is a primary cause for the reduced αSyn protein expression. We showed that expression ratios between mαSyn, hαSyn and hαSyn^*TP*^, or hαSyn^Δ119^ in the hippocampus, striatum, SN and olfactory bulbs are very similar, although the absolute protein content varied among the different brain regions.

Our models may identify differences between fibril- and oligomer-forming variants of αSyn, although we acknowledge that the present work does not experimentally investigate whether the two mutant αSyn give rise to two different types of aggregates. Previous work has indicated that hαSyn^TP^ favors oligomers while C-terminal truncations favor fibrillization (Crowther et al., 1998; Karpinar et al., 2009; Lazaro et al., 2014; Sorrentino and Giasson, 2020). hαSyn^Δ119^ is found in human synucleinopathies (Sorrentino and Giasson, 2020) and there is ample evidence showing that truncated hαSyn produces a strong phenotype when expressed in DAergic or forebrain neurons (Tofaris et al., 2006; Daher et al., 2009; Hall et al., 2015; Wegrzynowicz et al., 2019). hαSyn^TP^ was designed to hinder the efficient assembly of monomers into fibrils and it was shown to impair dopamine-related behavior in worms (*C. elegans*) and fruit flies (*D. melanogaster*; Karpinar et al., 2009). Worms typically slow down and reduce their search area in response to the presence of food; this behavior was impaired in worms expressing hαSyn^TP^ (Karpinar et al., 2009). Flies expressing hαSyn^TP^ were impaired to climb up an inverted vial in response to light, revealing motor defects (Karpinar et al., 2009). Cell-based studies corroborated hαSyn^*TP*^’s higher oligomerization and lower aggregation propensities in human cells (Lazaro et al., 2014). Here, we analyzed the pathophysiological consequences of hαSyn^TP^ in a vertebrate organism. By direct comparison of the two models, we identified features that are specific to each αSyn variant. We found many commonalities but also some differences. Both models showed late stage astro- and microgliosis to a similar extent, olfaction was impaired in both mutants and rotarod experiments revealed similar motor deficits in both models. Neither of the models showed loss of DAergic neurons and DA levels were similar to WT. The differences between hαSyn^TP^ and hαSyn^Δ119^ became apparent in the pole test, the Catwalk gait analysis and in compulsive behavior as revealed by the nestlet-shredding assay. It is interesting that the hαSyn^TP^ and hαSyn^Δ119^ mice exhibit such robust motor deficits although we detected neither DAergic cell loss nor reduction of DA concentration. In the hαSyn^Δ120^ mouse, motor deficits were shown to arise from synaptic dysfunction occurring before neuronal loss. Therefore, the motor deficits in our models could also arise from synaptic dysfunction in agreement with this earlier publication (Wegrzynowicz et al., 2019).

The pro-inflammatory cytokines (TNF-α and Il-6) were not altered in either *hαSyn^*TP*^* or *hαSyn^Δ119^* mice (Supplementary Figure 5), although both lines showed increased astrocyte and microglia reactivity (Figures 2A–D). In addition, the anti-inflammatory cytokine Il-10 and the chemokines Cxcl1, Ccl2, Ccl3, and Ccl4 were greatly up-regulated, especially in *hαSyn^Δ119^* mice. It is well documented that cytokine function, often duplicitous, varies depending on expression location, dosage, age and microenvironment, as they are complex molecules with far more than basic signaling inflammatory roles (Bourgognon and Cavanagh, 2020). Our observations in *hαSyn^*TP*^* and *hαSyn^Δ119^* mice show a definitively altered immunoactive state. Whether this is in indeed neuroinflammation and these molecules act in response to a neurotoxic effect, or are neuroprotective, would require further detailed investigation. Importantly, the underlying mechanism driving this immunoactive state seems to be different for each line, as chemokines were only significantly altered in *hαSyn^Δ119^* mice.

Other models have been developed in which truncated hαSyn was expressed under the control of the TH promoter (Tofaris et al., 2006; Daher et al., 2009; Wegrzynowicz et al., 2019). In two cases, hαSyn^Δ120^ was expressed in a mαSyn-deficient background (Tofaris et al., 2006; Wegrzynowicz et al., 2019). In another case, truncated hαSyn^Δ119^ was inserted downstream of a floxed-neo-polyA cassette and into the ROSA 26 locus (Daher et al., 2009). hαSyn^Δ119^ was then expressed in a Cre recombinase-dependent manner using TH-Cre mice (Daher et al., 2009). Truncated hαSyn^Δ120^ expression has also been directed to forebrain areas using the CamKII promoter (Hall et al., 2015). In the absence of knowledge of the cellular concentration of the corresponding αSyn C-terminal truncations, a side-by-side comparison between these and our models has to be interpreted with caution. Nonetheless, there are common features. Microgliosis was shown by CD11b immunoreactivity in the SN of mice expressing hαSyn^Δ120^ under the TH promoter (Tofaris et al., 2006). Consistent with that, we detected a strong inflammatory response in both our models by quantifying GFAP^+^ and Iba1^+^ cells. Similar to our models, loss of DAergic neurons was not observed in the SN of mice expressing hαSyn^Δ119^ in a Cre-dependent manner under the TH promoter (Daher et al., 2009), or hαSyn^Δ120^ under the TH promoter (Tofaris et al., 2006). In a separate study, however, the latter showed progressive TH^+^ cell loss starting at 9 months of age, becoming significant at 1 year of age and reaching a 54% reduction in these (MI2) mice by 20 months of age (Wegrzynowicz et al., 2019). Contrary to our results, the published lines show lower concentration of DA and its metabolites in the striatum (Tofaris et al., 2006; Daher et al., 2009; Wegrzynowicz et al., 2019). A gradual decline in rotarod performance was shown in MI2 mice starting at 1 year of age and becoming significant by 20 months of age (Wegrzynowicz et al., 2019). Both our mouse models showed a strong motor impairment in the rotarod. Transgenic mice expressing hαSyn^Δ120^ under the CamKII promoter showed an early and transient deficit in the buried food olfactory test (Hall et al., 2015). Both our models showed a strong olfactory impairment. Although we cannot rule out the possibility that motor impairment or motivation could contribute to the performance of mice in this task.

Our hαSyn^TP^ knock-in mice are a good example for how a mutation that may perturb protein aggregation can be introduced into the mammalian genome. Importantly, despite having sub-physiological levels of expression, hαSyn^TP^ mice indeed develop a late and progressive phenotype, which is as strong as that seen in the truncated models. hαSyn oligomers and fibrils have common and specific phenotypic features. Perhaps effective therapeutics are more likely to arise from strategies directed at both hαSyn species, such as anle138b (Wagner et al., 2013).

One feature of the *hαSyn^*tm1*^* and *hαSyn^*tm2*^* strains is that the WT *hαSyn* is flanked by loxP sites. Therefore, these lines can be crossed to a wide spectrum of Cre-drivers (i.e., inducible Dat-Cre), providing a more fine-tuned spatio-temporal control of mutant hαSyn expression. The downside of such knock-in strategies and ability for sophisticated regulation may be that the presence of transgenic DNA sequences and of regulatory DNA elements in the murine *αSyn* locus influence the level of *hαSyn* expression. A remedy of this problem could be the introduction of ectopic moderate enhancers into the targeting vector. Our work could inspire the creation of knock-in models of modified proteins that lead to abnormal quaternary structures. Resulting mice may serve as sources of iPSCs from which, e.g., brain organoids can be created for mechanistic and therapeutic studies.

## Materials and Methods

### Generation of the *hαSyn^*TP*^* and *hαSyn^Δ119^* Mouse Lines

The genomic locus of murine αSyn and the targeting vector are shown in Supplementary Figures 1A,B. The two knock-in lines differed only in the cassette encoding the hαSyn mutant protein. hαSyn^TP^ carried three alanine to proline substitutions at residues 30 (GCA to CCA), 56 (GCT to CCG), and 76 (GCA to CCG). The C-terminal truncated hαSyn terminated with aspartic acid at residue 119 (120 CCT to TAA). Conditional alleles were generated using standard recombineering methods resulting in the targeted allele shown in Supplementary Figure 1C. Briefly, we constructed a targeting vector consisting of homologous arms, loxP-flanked *hαSyn*, *hαSyn^*TP*^* or *hαSyn^Δ119^*, a *pLAP* reporter, and selectable markers (Supplementary Figure 1B). The homologous arms of the targeting vector were 5.4 kb and 3.4 kb, respectively. We targeted exon 3 of the *mαSyn* gene, thus placing the transgenes under the endogenous promoter and disrupting the murine αSyn (Supplementary Figure 1A). The insert displaced exons 3 to 7 downstream. However, these exons did not give rise to mαSyn (Supplementary Figures 2, 3).

Transfection into 129/SvJ ES cells and generation of heterozygous founder mice were carried out by the Institute’s animal facility. The FRT-flanked PGK-neo selection cassette was removed by crossing mice with a Flippase expressing deleter line (Farley et al., 2000). This generated the *hαSyn^*tm1*^* line (targeted mutation 1, Figure 1A, blue mouse). To generate the *hαSyn^*TP*^* line, we crossed *hαSyn^*tm1*^* to CMV-Cre mice (Schwenk et al., 1995). These mice were backcrossed to a pure C57BL/6J genetic background for at least 9 generations. To generate experimental and control littermate mice we mated *hαSyn^*TP*/+^* to *hαSyn^*TP*/+^* and *hαSyn^*tm1*^*^/+^ to *hαSyn^*tm1*^*^/+^ mice. For the *hαSyn^Δ119^* line, we proceeded in an analogous manner. Primers for genotyping were 5′ – TGA CAT GAC TTT TTC CTA GTA TTG AG – 3′ (Primer 1), 5′ – AGA TGT ATT TTT GCT CCA CAC TAG – 3′ (Primer 2), and 5′ – CCT GGG GTT CGT GTC CTA C – 3′ (Primer 3). Primers were used in two separate PCR reactions with primers 1 + 2 or primers 1 + 3 (Figure 1A and Supplementary Figures 1A,C,E,F). Primers 1 + 2 generated a 256 bp PCR product from the WT allele or a 366 bp product from the floxed-*hαSyn* allele (Supplementary Figures 1E,F). Primers 1 + 3 generated a 225 bp PCR product from the mutant allele (Supplementary Figures 1E,F).

### Mass Spectrometry

#### Chemicals

For LC/MS sample preparation, LC/MS-grade water and acetonitrile (ACN) were used, if not otherwise stated, and were purchased together with acetone from Merck, Darmstadt, Germany. Triethylammonium bicarbonate buffer (TEAB), formic acid (FA), EDTA tris(2 carboxyethyl)phosphine (TCEP) and iodoacetamide (IAA) and trifluoroethanol (TFE) were purchased from Sigma-Aldrich, Taufkirchen, Germany. SDS was purchased from Serva Electrophoresis GmbH, Heidelberg, Germany. TFA was obtained from Roth, Karlsruhe, Germany. Rapigest was obtained from Waters, Milford, United States. ^15^N-labeled αSyn was purchased from rPeptide, Watkinsville, United States. MS-grade trypsin and LysN were obtained from Promega, Madison, United States. TMT10plex and TMT11-131C were purchased from Thermo Fisher Scientific, Bleiswijk, Netherlands.

#### Protein Extraction and Digestion

Tissue samples from hippocampus, striatum, olfactory bulb, and SN of 1.5 year-old mice were weighed out and homogenized in 10 μL of lysis buffer (4% SDS, 1 mM EDTA in 100 mM HEPES, pH 8) per 1 mg of wet tissue. Homogenization was performed using 12 glass/zirconium beads and 3 cycles in FastPrep-24 homogenizer (MP Biomedicals, Eschwege, Germany) at 6.5 m/s for 20 s followed by 10 min sonication in the Bioruptor (Diagenode, Seraing, Belgium) using 15 s on/15 s off cycles at maximum output. Protein concentration was assessed using BCA protein assay kit (Thermo Fisher Scientific, Waltham, United States) according to the manufacturer’s instructions. 1 mg total protein was treated with 10 mM TCEP for 30 min at 55°C and alkylated using 18.75 mM IAA for 20 min at RT in the dark. Afterward, proteins were precipitated with 80% (v/v) acetone and kept overnight at –20°C. Protein pellets were washed twice with ice-cold 80% (v/v) acetone/water and re-dissolved in 1% (w/v) Rapigest in 100 mM TEAB. Protein concentration was estimated using BCA protein assay kit as described above. Equal protein amounts were diluted 10 times with 100 mM TEAB and subjected for endoproteinase digestion using sequencing grade trypsin at 1:20 trypsin-to-protein ratio (w/w). Prior to digestion, 200 ng of ^15^N-labeled αSyn were added to each sample. Digestions were carried out overnight at 37°C. Alternatively, proteins were digested using LysN at 1:100 protein-to-protease ratio (w/w). Afterward, RapiGest was hydrolyzed by incubating with 1% (v/v) TFA at 37°C for 1 h and non-soluble fragments removed by centrifugation. Peptide samples were dried in a centrifugal Savant SpeedVac vacuum concentrator (SpeedVac, Thermo Fisher Scientific, Waltham, United States), dissolved in 2% (v/v) ACN, 0.1% (v/v) TFA in water and subjected to LC-MS/MS analysis.

#### LC-MS/MS Analysis: Single Reaction Monitoring

To assess expression of αSyn, each sample was injected in technical duplicate into UltiMate 3000 RSLC nanosystem (Thermo Fisher Scientific, Waltham, United States) equipped with a C18 PepMap100 Precolumn (0.3 × 5 mm, 5 μm, Thermo Fisher Scientific) and an in-house packed C18 analytical column (75 μm × 300 mm; Reprosil-Pur 120C18-AQ, 1.9 μm, Dr. Maisch GmbH, Ammerbuch, Germany). Tryptic and LysN-generated peptides from hippocampal samples were eluted using a linear gradient ranging from 10 to 42% of mobile phase B [80% (v/v) ACN, 0.1% (v/v) FA in water] over 43 min. Tryptic and LysN-generated peptides from striatum, olfactory bulb, and SN were eluted using linear gradient from 7 to 23.5% of mobile phase B over 15 min followed by increase to 28% B over next 15 min. Eluting peptides were sprayed into a triple quadrupole mass spectrometer (Quantiva, Thermo Fisher Scientific, Bremen, Germany) operated in a single reaction monitoring mode. Cycle time was set to 1.6 s, Q1 and Q3 resolution to 0.7. Transition ions of the following αSyn peptides (light and ^15^N-labeled, when applicable) were monitored: EGVVHGVATVAEK, EQVTNVGGAVVTGVTAVAQK, TVEGAGSIAAATGFVK, EG VVHGVTTVAEK, TVEGAGNIAAATGFVK, EGVVHGVATVP EK, EQVTNVGGAVVTGVTPVAQK, and KNEEGAPQEGIL EDMPVD (LysN). Transition intensities were extracted from the raw data using Skyline (version 19.1.0.193; MacLean et al., 2010) and further analyzed using in house written R scripts^1^. Specifically, intensities of the six most intense transition ions were summed per precursor ion. Intensities of the ^15^N-labeled αSyn peptides were used to derive normalization factors as well as to estimate the absolute αSyn amount per sample. For each peptide, the linear relationship between peptide intensity and protein concentration was confirmed using dilution series of ^15^N-labeled αSyn digested with trypsin and spiked into the tissue lysates. The lowest limit of quantification was estimated from the graph (Supplementary Figure 6) based on a double limit of detection calculated using MSstats (Galitzine et al., 2018).

#### Data Availability

The MS data have been deposited to the ProteomeXchange Consortium via the PRIDE partner repository with the dataset identifier PXD022314 (SRM experiments).

### RNA Isolation, PCR and qPCR

Total RNA was extracted from fresh frozen tissue using the RNeasy Mini Kit (Qiagen, Hilden, Germany, Cat # 74104) according to the manufacturer’s instructions. RNA extracts were quantified using a spectrophotometer and 1 μg RNA was reverse transcribed using the QuantiTect Reverse Transcription Kit (Qiagen, Cat. # 205313) according to the manufacturer’s protocol. cDNA samples were subsequently used for PCR or qPCR.

Primers used for RT-PCR amplifying hαSyn from exon2 to the polyA were 5′ – GTT CTT CAG AAG CCT AGG GAG – 3′ (hαSyn Fo) and 5′ – CTG TCA GCA GAT CTC AAG AAA C – 3′ (hαSyn Re) and yielded a 512 bp PCR product. Primers amplifying hαSyn^TP^ from exon 2 to the IRES cassette were 5′ – GTT CTT CAG AAG CCT AGG GAG – 3′ (hαSyn^TP^ Fo) and 5′ – GAT ACG CGT ACG TCG CGA CC – 3′ (hαSyn^TP^ Re) and yielded a 566 bp product. Primers amplifying hαSyn^Δ119^ from exon 2 to the IRES cassette were 5′ – GTT CTT CAG AAG CCT AGG GAG – 3′ (hαSyn^Δ119^ Fo) and 5′ – GAT ACG CGT ACG TCG CGA CC – 3′ (hαSyn^Δ119^ Re) and yielded a 503 bp product.

For qPCR, samples were diluted 1:20 and 5 μl cDNA were used. We included three mice per group and two technical replicates per mouse. qPCR was performed using iQ^TM^ SYBR^®^ Green Supermix (BioRad, Hercules, United States, Cat. # 170-8886) on an iCycler CFX96 Real-Time PCR Detection System thermocycler (BioRad). Primers used were 5′ – AAG AGG GTG TTC TCT ATG TAG GC – 3′ and 5′ – GCT CCT CCA ACA TTT GTC ACT T – 3′ for hαSyn (either WT or mutant). Supplementary Table 6 lists primers sequences used for pro-inflammatory cytokines. Gapdh was used as housekeeping control for normalization with 5′ – CAT GGC CTT CCG TGT TCC TA – 3′ and 5′ – CCT GCT TCA CCA CCT TCT TGA – 3′ primers (Urban et al., 2019). Relative quantification of expression was performed using the ΔΔCT method as previously described (Livak and Schmittgen, 2001).

### Immunohistochemistry

Coronal, 10 μm thick, cryosections from perfused mice (with ice-cold PBS followed by 4% PFA in PBS) were equilibrated to room temperature for 10 min before use.

#### Immunofluorescence

Sections were hydrated with 3 washes of 1 × PBS for 2 min and treated with Triton-X (0.25% Triton-X in PBS) for 10 min. Sections were then blocked (5% normal goat serum, 5% BSA, 0.25% Triton-X in PBS) for 1 h at room temperature and incubated overnight with primary antibody in blocking solution at 4°C (GFAP, 1:500, abcam 4674, Abcam, Cambridge, United Kingdom; Iba1, 1:1000, Synaptic Systems 234-003, Göttingen, Germany). On the following day, sections were washed 6 times in 1 × PBS+T_20_ (0.1 % Tween-20) for 5 min and incubated at room temperature for 1 h with secondary antibodies conjugated to Alexa flours (Invitrogen, Carlsbad, United States) diluted 1: 2000 in blocking solution. Six final washes of 6 min each in 1 × PBS+T_20_ were done before sections were coverslipped in Vectashield Antifade Mounting Medium with DAPI (Vector Labs, Burlingame, United States). Three striatal fields were captured per hemisphere (as in Figure 2B) using an inverted fluoresce microscope (Leica DMI6000B, Leica, Wetzlar, Germany). GFAP^+^ and Iba1^+^ cells were counted using the ImageJ particle analysis tool (Schneider et al., 2012). We included 3 mice per group, 4 striatal sections at Bregma – 0.10 mm per mouse and 6 captured striatal fields per section.

#### Chromogenic Immunostaining

Sections were hydrated with 2 washes of 1 × PBS for 2 min each before they underwent antigen retrieval (3 min boiling in microwave in 10 mM Na-Citrate buffer pH 6.0 followed by 30 min cooling at room temperature). Sections were washed twice in 1 × PBS for 2 min, endogenous peroxidase was quenched with 0.6% H_2_O_2_ in PBS for 30 min at room temperature and sections were washed again 3 times in 1 × PBS for 2 min each. Sections were blocked in 5% normal goat serum, 5% BSA, 0.25% Triton-X in PBS for 1 h at room temperature. Overnight incubation with primary antibody in blocking solution followed at 4°C (Th, 1:2000, Millipore MAB318, Merck Millipore, Burlington, United States). Th-immunostainned sections were visualized by chromogenic stain using Vectastain Elite ABC-kit with peroxidase (Vector Labs, Cat. # PK-6102) and ImmPACT DAB peroxidase substrate (Vector Labs. Cat. # SK-4105) according to the manufacturer’s instructions. The desired stain intensity was developed by 3 sequential incubations in peroxidase substrate solution DAB for 20, 30, and 40 min each. Finally, sections were rinsed in water and coverslipped with HydroMarix medium (Micro-Tech-Lab, Graz, Austria).

### Stereologic Quantification of Substantia Nigra Neurons

The number of Th-positive neurons in the SN was stereologically assessed as previously described (Tonges et al., 2012; Tatenhorst et al., 2014; Balke et al., 2020) by a blinded investigator. Every 10th section through the SN (section cut thickness: 10 μm, counted sections per animal: 12) was analyzed using Stereo Investigator software (Version 2019; MBF Bioscience, MicroBrightField Inc., Williston, VT, United States) on a Zeiss AxioImager M2 microscope equipped with a Zeiss 506 AxioCam camera (Zeiss, Göttingen, Germany). A 2.5x objective was used to outline the respective areas of the SN pars compacta. Th-positive cells were counted using a 40x objective. The counting frame size was 50 × 50 μm, the grid size was 100 × 100 μm. The number of Th-positive cells was finally calculated by the optical fractionator method of the Stereo Investigator software as described before (Tonges et al., 2012; Tatenhorst et al., 2014; Balke et al., 2020). Values represent counts of one unilateral SN.

### HPLC

Analysis of dopamine (DA) and its metabolites was performed as previously described (Tonges et al., 2012; Tatenhorst et al., 2014; Vingill et al., 2016; Balke et al., 2020). Tissue samples were homogenized using 1.4 mm ceramic beads in a bead mill homogenizer (Precellys 24; Peqlab, Erlangen, Germany) with 50 μL 0.1 mol/l perchloric acid (Merck, Darmstadt, Germany) per milligram of striatal tissue. Homogenates were centrifuged at 4°C and 12 000 RPM for 5 min and supernatants were centrifuged again for 10 additional minutes. 20 μl of supernatant were injected onto a C18 reverse-phase HR-80 catecholamine column (ESA, Bedford, United States). DA, 3,4-DOPAC, and HVA were quantified by high-performance liquid chromatography (HPLC) with electrochemical detection. The mobile phase (pH 4.3) consisted of 6.9 g/l of sodium acetate (Carl Roth, Karlsruhe, Germany), 48 mg/l of EDTA (Applichem, Darmstadt, Germany), 7.3 g/l of citric acid (Sigma, Taufkirchen, Germany), 105 mg/l of octane sulfonic acid (Fluka, Seelze, Germany), and 10% methanol (Applichem). The flow rate was kept constant at 0.4 ml/min. Samples were run alternately and DA, HVA, and DOPAC standards (in concentrations of 0.15, 0.3, and 1.5 μM) were run at regular intervals between the samples to ensure accurate measurement of the catecholamines. Peaks were detected by an ESA Coulochem III with a model 5010 detector (E1 = 50 mV; E2 = 400 mV). Data were collected and processed using a Chromeleon computer system (Dionex, Idstein, Germany). Finally, the area under the specific peak was analyzed to determine the concentration of DA, DOPAC and HVA in ng per mg wet tissue.

### Mouse Experiments

Animal care and handling were carried out in full compliance with the Declaration of Helsinki. All experiments were approved by the Lower Saxony State Office for Consumer Protection and Food Safety and performed in accordance with the German Law of Animal Welfare.

All mice were maintained on a C57BL/6J background. They were kept in a 12 h light/dark cycle and housed in groups under constant standard conditions of temperature and humidity. Mice had *at libitum* access to food and water, unless otherwise noted. Only male mice were used for this study. Mice selected for behavior experiments were single-caged and brought into the testing rooms at least 7 days prior to the beginning of the experiment. At 1 year of age, mice underwent nestlet-shredding and pole testing. At 18 months of age, they underwent a battery of motor and non-motor tests. Subsequently, histochemical and biochemical analyses were performed. Mice had a rest period of at least 3 days in between tests. Initial groups consisted of 25 mice per genotype. By 1 year of age, groups ranged from 17 to 25 mice. As mice aged, due to illness or death, group sizes at 1.5 years ranged from 14 to 19.

For molecular and biochemical analyses, mice were sacrificed by cervical dislocation, brains were quickly removed from the skull, regions of interest were isolated on ice, tissue was fresh frozen in liquid nitrogen and stored at –80°C until further use. The SN and VTA were microdissected as previously described (Salvatore et al., 2012). Briefly, we placed the brain upside-down in a pre-cooled stainless steel coronal mouse brain matrix and took the next six to seven 1 mm-thick sections caudal of the optic chiasm. The hippocampus was also microdissected from the sections above containing the SN and VTA. For histological analysis, mice were deeply anesthetized and transcardially perfused with ice-cold PBS followed by 4% PFA in PBS. Brains were quickly removed from the skull and sucrose protected before they were embedded in OCT. Coronal cryosections 10 μm -thick were sectioned, transferred to Superfrost slides and stored at –20°C until further use.

#### Buried Food Test

The buried food test was adapted from previously described protocols (Yang and Crawley, 2009; Hall et al., 2015) with the following modifications. Briefly, mice were placed in experimental cages once daily for 2 consecutive days and they were allowed to explore undisrupted for 30 min before returning to their home cage. Experimental cages consisted of bedding material filled to approx. 3 cm-deep and a cage lid. On day 3, chow was removed from the home cages at least 6 h prior to the experiment to assure enough motivation to search for food. Mice were then placed into their experimental cage and allowed to habituate for 30 min. Thereafter, a Choco puff^®^ ball was buried 1 cm deep into the bedding material at the opposite side (relative to the position of the mice) and the time to find the ball was recorded. The timer was stop as soon as mice placed at least one paw and their snout onto the ball. Mice were allowed up to 3 min to fulfill the task using only their sense of smell. A maximum score of 180 s was assigned to mice unable find the cereal ball.

#### Nestlet-Shredding Test

Nestlet-shredding test was performed as previously described (Witkin, 2008) with minor modifications. Nesting material available in home cages was first removed. Then, 3 pre-weighed cotton nestlet pads were placed onto the cage’s metallic grid lid. We weighed the remaining nesting material exactly after 24, 48, and 72 h after.

#### Vertical Pole Test

The vertical pole test was carried out as previously described (Ogawa et al., 1985; Matsuura et al., 1997; Hölter and Glasl, 2012) with some modifications. Mice were trained to climb down a rough-surfaced vertical pole (50 cm, 1 cm diameter). Mice were placed head-upward on the top and if not immediately turning, they were manually helped. Once they reached the floor, they were immediately returned to their home cage. On days 1 and 2, mice were allowed 3 training trials per day with a resting period of at least 10 min in between trials. On day 3, mice underwent 5 testing trials with a resting period of at least 10 min in between trials. We recorded the time it took mice to turn around and descend the pole. Time was stopped when at least one of the front paws touched the bedding material and falls were scored with the max value of 60 s. Only the three best trials were considered for analysis of group differences in time to descend. To calculate the percentage of mice falling, all trials were considered.

#### Rotarod Test

Mice were trained over two consecutive days. Each training day consisted of two trials, 6 h apart. Mice were trained on a rotarod (Ugo Basile) rotating at a constant speed of 10 rpm for 180 s. Mice falling were carefully placed back onto the rod. Mice were then tested on the rotarod gradually accelerating 5–40 rpm for a maximum of 180 s over 2 days with two trials per day and 6 h in between trials. Time (latency) to fall was recorded. Because weight could affect performance (McFadyen et al., 2003), mice were weighed prior to the experiment.

#### CatWalk Gait Analysis

Catwalk XT gait analysis system (Noldus, Wageningen, Netherlands) was used to monitor gait performance as previously described (Saal et al., 2015; Tatenhorst et al., 2016; Balke et al., 2020). Briefly, animals were placed in a walkway 4 cm wide and videotaped from below. Footprints were automatically detected by the Catwalk XT 10.0 gait analysis software. Detection settings were as set as follows: camera gain 20, intensity threshold 0.10, max. allowed speed variation 60%. Three compliant runs per animal were recorded and means out of these runs were analyzed with Catwalk XT 10.0 gait analysis software.

### Statistical Analysis

Unless specifically mentioned otherwise, data were analyzed by One- and Two-way ANOVA (Analysis of Variance) with repeated-measures, Tukey’s or Bonferroni’s post-test for multiple comparisons when appropriate (see Supplementary Tables for specific analysis and post-test used in each data set). Errors are displayed as standard error of the mean. Data analysis was performed using the GraphPad Prism 8.3.0 (GraphPad Software, San Diego, CA, United States).

## Data Availability Statement

The datasets presented in this study can be found in online repositories. The names of the repository/repositories and accession number(s) can be found below: https://github.com/IvanSilbern/2021_AMartinez_aSyn, in-house written R scripts; http://www.proteomexchange.org/, PXD022314.

## Ethics Statement

The animal study was reviewed and approved by Lower Saxony State Office for Consumer Protection and Food Safety (LAVES).

## Author Contributions

AM designed cloning strategy, generated the mouse models, performed experiments, analyzed results, coordinated the study, and wrote the manuscript. IS performed mass spectrometry experiments and analyzed results. IG designed cloning strategy and generated the mouse models. LT performed HPLC experiments and stereological counting, analyzed results, and provided support for CatWalk experiments. SB generated recombinant synuclein. HU supervised mass spectrometry experiments. MZ and CG supervised study. GE designed and supervised study and wrote the manuscript. All authors reviewed, discussed and approved the manuscript.

## Conflict of Interest

MZ and CG are inventors on WO/2010/015714 that described the properties of the triple proline mutant. CG is shareholder of the company MODAG, which aims to treat neurodegenerative diseases. The remaining authors declare that the research was conducted in the absence of any commercial or financial relationships that could be construed as a potential conflict of interest.

## Footnotes

1 Available at https://github.com/IvanSilbern/2021_AMartinez_aSyn
