# Supplementary material for: Low-Expressing Synucleinopathy Mouse Models Based on Oligomer-Forming Mutations and C-Terminal Truncation of α-Synuclein
Source: Front Neurosci. 2021 Jun 17;15:643391. doi: 10.3389/fnins.2021.643391 (PMC8248494; doi:10.3389/fnins.2021.643391)
Supplement: Supplementary file 1 [file Image_1.pdf]

Supp. Fig. 1

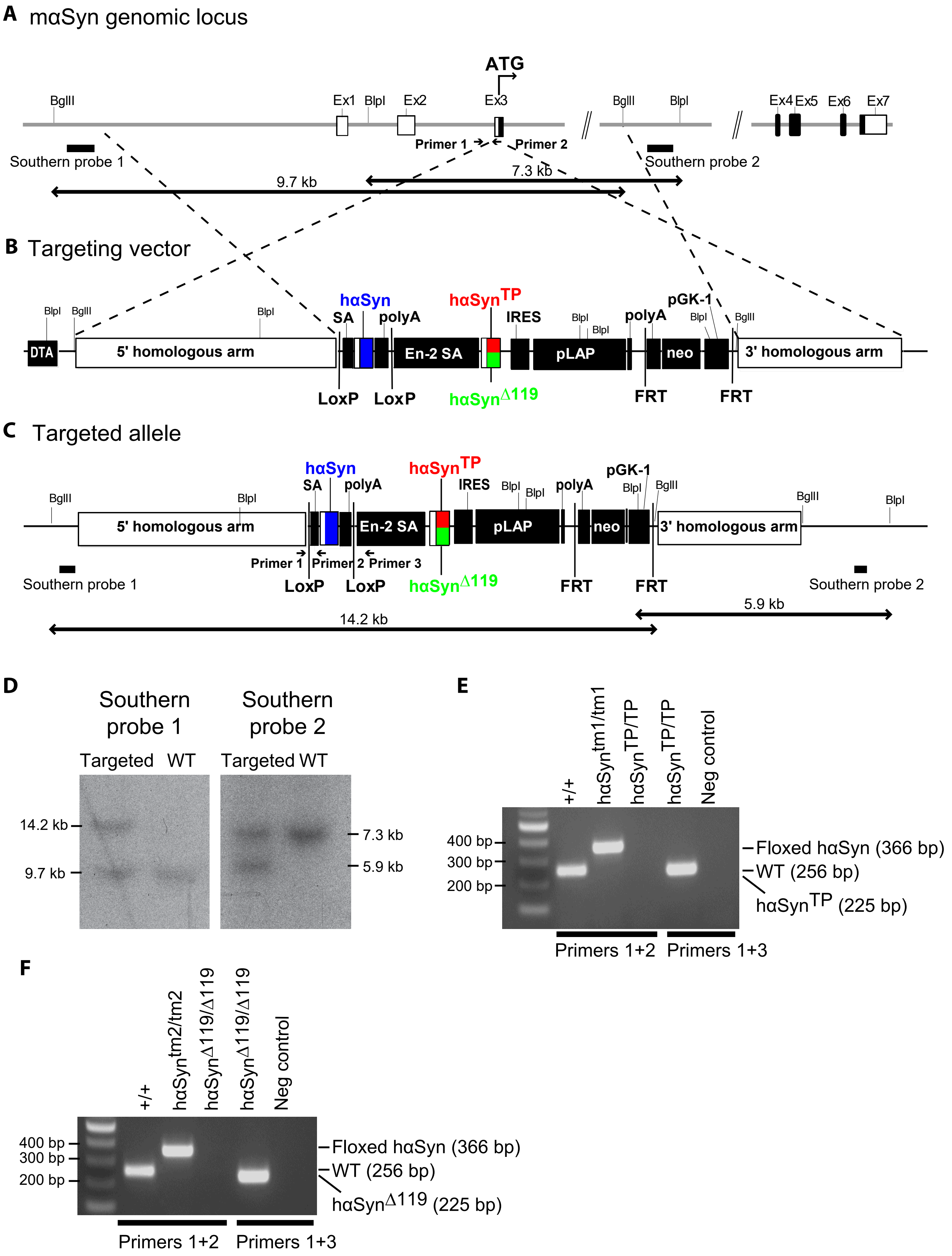
A mαSyn genomic locus
ATG
BglII
Ex1
BlpI
Ex2
Ex3
BglII
BlpI
Ex4
Ex5
Ex6
Ex7
Primer 1
Primer 2
Southern probe 1
Southern probe 2
7.3 kb
9.7 kb
B Targeting vector
DTA
BlpI
BglII
5' homologous arm
BlpI
hαSyn
SA
polyA
En-2 SA
hαSynTP
IRES
BlpI
BlpI
pLAP
polyA
pGK-1
BlpI
BglII
neo
3' homologous arm
LoxP
LoxP
hαSynΔ119
FRT
FRT
C Targeted allele
BglII
5' homologous arm
BlpI
hαSyn
SA
polyA
En-2 SA
hαSynTP
IRES
BlpI
BlpI
pLAP
polyA
pGK-1
BlpI
BglII
neo
3' homologous arm
BglII
BlpI
Primer 1
Primer 2
Primer 3
Southern probe 1
LoxP
LoxP
hαSynΔ119
FRT
FRT
Southern probe 2
5.9 kb
14.2 kb
D
Southern probe 1
Southern probe 2
Targeted
WT
Targeted
WT
14.2 kb
9.7 kb
7.3 kb
5.9 kb
E
+/+
hαSyntm1/tm1
hαSynTP/TP
hαSynTP/TP
Neg control
400 bp
300 bp
200 bp
Floxed hαSyn (366 bp)
WT (256 bp)
hαSynTP (225 bp)
Primers 1+2
Primers 1+3
F
+/+
hαSyntm2/tm2
hαSynΔ119/Δ119
hαSynΔ119/Δ119
Neg control
400 bp
300 bp
200 bp
Floxed hαSyn (366 bp)
WT (256 bp)
hαSynΔ119 (225 bp)
Primers 1+2
Primers 1+3

Supp. Fig. 1. Detailed construct design and genotyping of the generated mouse lines. **A.** Murine *αSyn* genomic locus. Primers 1 and 2 were used for genotyping (see also **(C)** and **(E)**). Southern probes 1 and 2 used in Southern analyses to identify targeted ES cell clones. **B.** We targeted exon 3 of the mouse *αSyn*. The targeting vector included a floxed-wild type *hαSyn* (blue), either $h\alpha Syn^{TP}$ (red) or $h\alpha Syn^{\Delta 119}$ (green), a *pLAP* reporter and a *neo* cassette. Various regulatory sequences and homologous arms were also included. DTA: Diphtheria toxin A; En-2 SA: Engrail-2 splicing acceptor; IRES: Internal ribosomal entry site; pGK-1: phosphoglycerate kinase 1 promoter; neo: neomycin; pLAP: placental alkaline phosphatase reporter; SA: splicing acceptor. **C**. Targeted allele. Primers 1, 2 and 3 were used for genotyping (see **(E)** and **(F)**). Southern probes 1 and 2 used in Southern analyses to identify positive ES cell clones. **D.** Southern blots of positive clones using Southern probes 1 and 2. Southern probe 1 was used to identify genomic DNA digested with BglII. This enzyme generated a 9.7 kb fragment in the WT allele or a 14.2 kb fragment for the targeted allele, indicated by a thick horizontal line in **(A)** and **(C)**. Southern probe 2 was used to identify genomic DNA digested with BlpI. This enzyme generated a 7.3 kb fragment in the WT allele or a 5.9 kb fragment for the targeted allele, indicated by a thick horizontal line in **(A)** and **(C)**. Blots shown here are from the $h\alpha Syn^{tm1}$ line. Screening for the $h\alpha Syn^{tm2}$ line used the same strategy yielding diagnostic fragments of 14.2 kb and 5.9 kb for BglII and BlpI mutant DNA digests (not shown). **E.** PCR genotyping of $h\alpha Syn^{tm1}$ and $h\alpha Syn^{TP}$ mice required two PCR reactions. Primers 1 + 2 generated a 256 bp product for WT and a 366 bp product for the $h\alpha Syn^{tm1}$ allele. Primers 1 + 3 generated a 225 bp product in the $h\alpha Syn^{TP}$ allele. **F.** PCR genotyping of the $h\alpha Syn^{tm2}$ and $h\alpha Syn^{\Delta 119}$ mice required two PCR reactions. Primers 1 + 2 generated a 256 bp product for WT and a 366 bp product for the $h\alpha Syn^{tm2}$ allele. Primers 1 + 3 generated a 225 bp product in the $h\alpha Syn^{\Delta 119}$ allele.
