# Supplementary material for: Low-Expressing Synucleinopathy Mouse Models Based on Oligomer-Forming Mutations and C-Terminal Truncation of α-Synuclein
Source: Front Neurosci. 2021 Jun 17;15:643391. doi: 10.3389/fnins.2021.643391 (PMC8248494; doi:10.3389/fnins.2021.643391)
Supplement: Supplementary file 2 [file Image_2.pdf]

Supp. Fig. 2

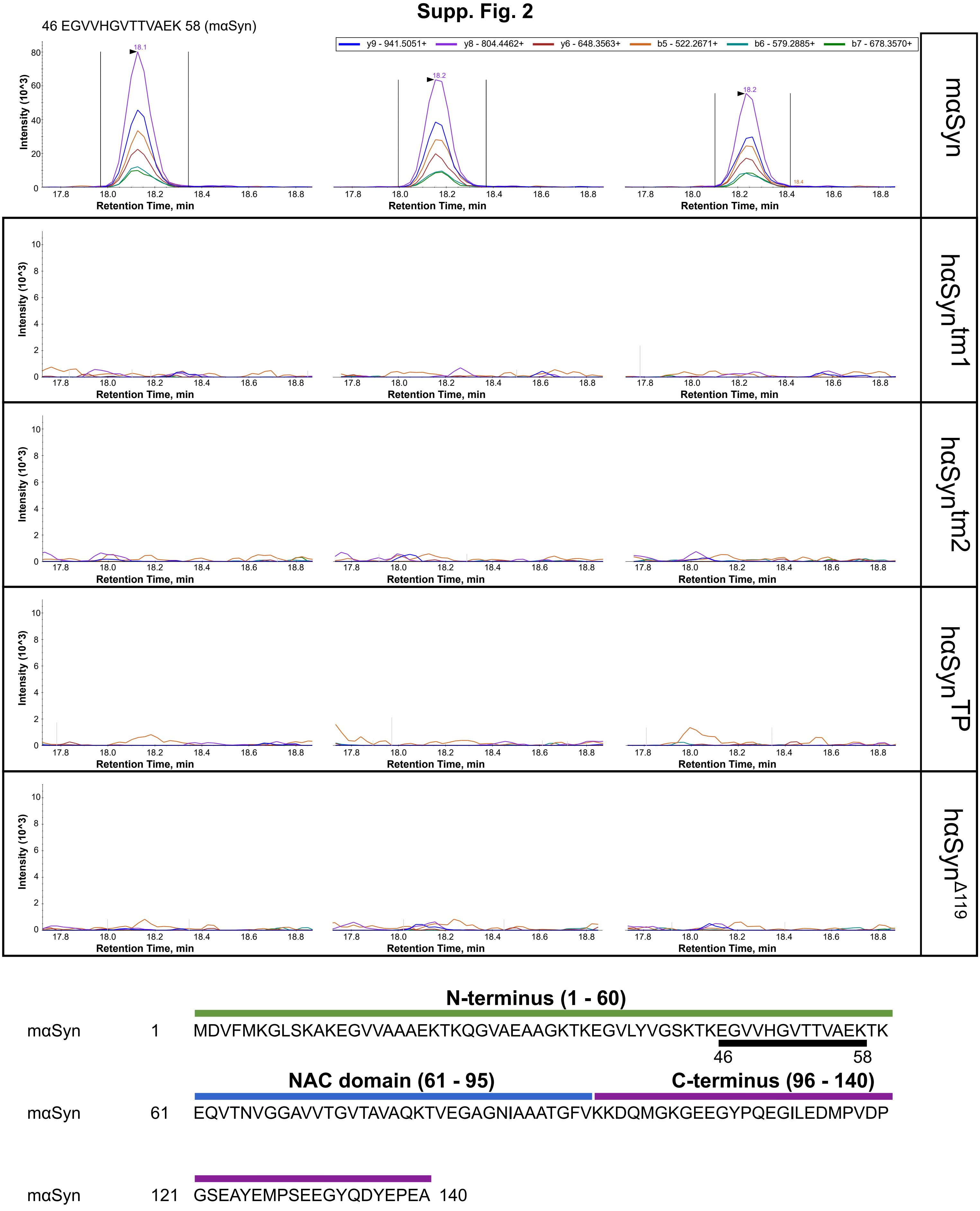

Supp. Fig. 2. Elution profiles of the six most-intense fragment ions of the mαSyn-specific peptide 46 - EGVV-HGVTTVAEK - 58. Each fragment ion was named as: "y" fragments containing C-terminus, or "b" fragments containing N-terminus, followed by the number of amino acids that the fragment ion consisted of and its corresponding mass (single-charged). We detected this peptide only in wild type mice, confirming that no partial mαSyn was produced in any of the transgenic lines. Note that this peptide is located in the N-terminus of the mαSyn protein (bottom diagram).
