# Supplementary material for: Low-Expressing Synucleinopathy Mouse Models Based on Oligomer-Forming Mutations and C-Terminal Truncation of α-Synuclein
Source: Front Neurosci. 2021 Jun 17;15:643391. doi: 10.3389/fnins.2021.643391 (PMC8248494; doi:10.3389/fnins.2021.643391)
Supplement: Supplementary file 4 [file Image_4.pdf]

Supp. Fig. 4

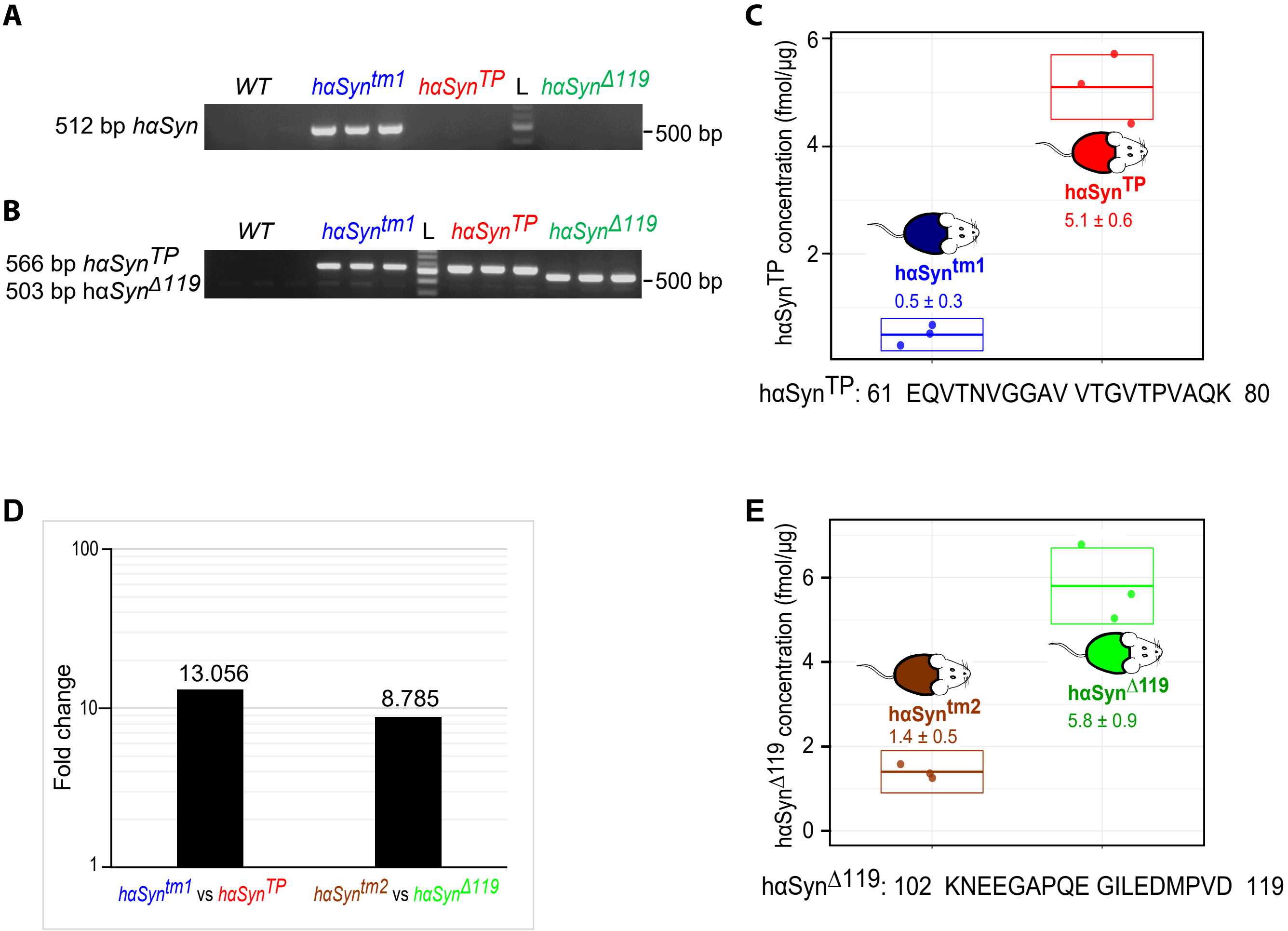

Supp. Fig. 4. Analysis of *αSyn* transcripts (A, B, D) and proteins (C, E). **A.** RT-PCR using primers (sequence in Material and Methods) designed to identify *hαSyn* produced the expected 512 bp band in *hαSyn*^{tm1} mice, but not in *hαSyn*^{TP} or *hαSyn*^{Δ119} mice. Data from cortical cDNA of 3 mice aged 1.5 years are shown. L: Ladder. **B.** RT-PCR using primers (Material and Methods) designed to identify *hα Syn*^{TP} produced the expected 566 bp amplicon in *hαSyn*^{TP} mice. This amplicon was also detected in *h αSyn*^{tm1} mice, and is the result of read-through transcription. Analogously, RT-PCR using primers identifying *hαSyn*^{Δ119} (sequence in Material and Methods) produced the expected 503 bp band in *hα Syn*^{Δ119} mice. Data from cortical cDNA of 3 mice aged 1.5 years are shown. L: Ladder. **C.** MS quantification of hαSyn^{TP} protein in 1.5 years old *hαSyn*^{tm1} mice using a hαSyn^{TP}-specific peptide (aa 61 - 80). We found an approximately 10-fold lower amount of hαSyn^{TP} protein in the parental strain when compared to age-matched *hαSyn*^{TP} mice. **D.** qPCR using *hαSyn* pan-primers (recognizing wild type or mutant *hαSyn*; sequence in Material and Methods) showed approximately 13 and 9 times more transgenic *hαSyn* transcripts in the parental *hαSyn*^{tm1} and *hαSyn*^{tm2} strains than mutant transcripts in their corresponding *hαSyn*^{TP} or *hαSyn*^{Δ119} lines. Age of mice was 1.5 years. **E.** MS quantification of hαSyn ^{Δ119} protein in 1.5 years old *hαSyn*^{tm2} mice using an hαSyn^{Δ119}-specific peptide (aa 102 – 119, full length αSyn would generate the aa 102 – 140 C-terminal peptide). We found hαSyn^{Δ119} protein in the parental strain but at approximately 4-fold lower level than in age-matched *hαSyn*^{Δ119} mice.
