# Supplementary figures and images for: Low-Expressing Synucleinopathy Mouse Models Based on Oligomer-Forming Mutations and C-Terminal Truncation of α-Synuclein
Source: Front Neurosci. 2021 Jun 17;15:643391. doi: 10.3389/fnins.2021.643391 (PMC8248494; doi:10.3389/fnins.2021.643391)

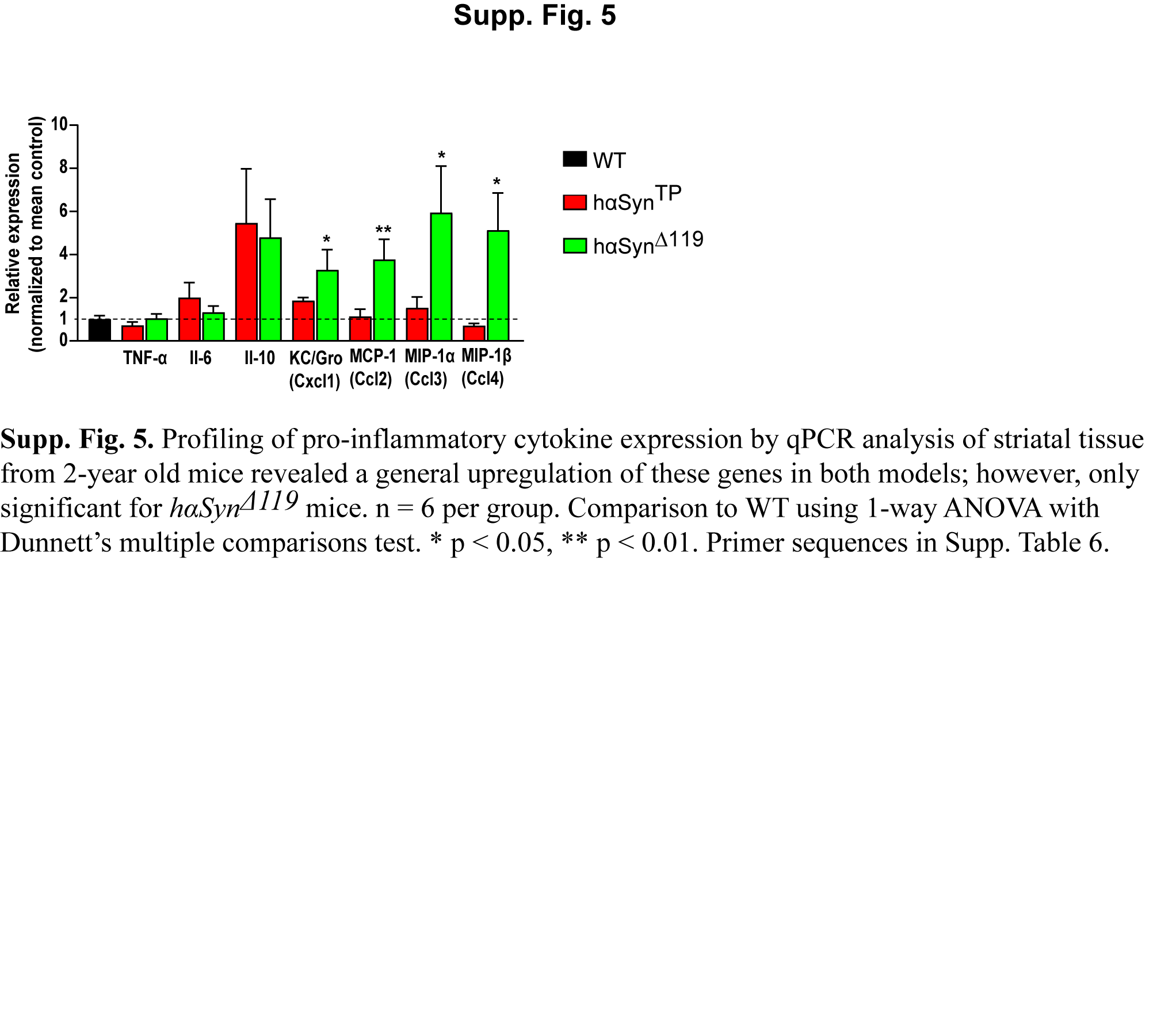

Supplement: Supplementary file 5 [file Image_5.tif]

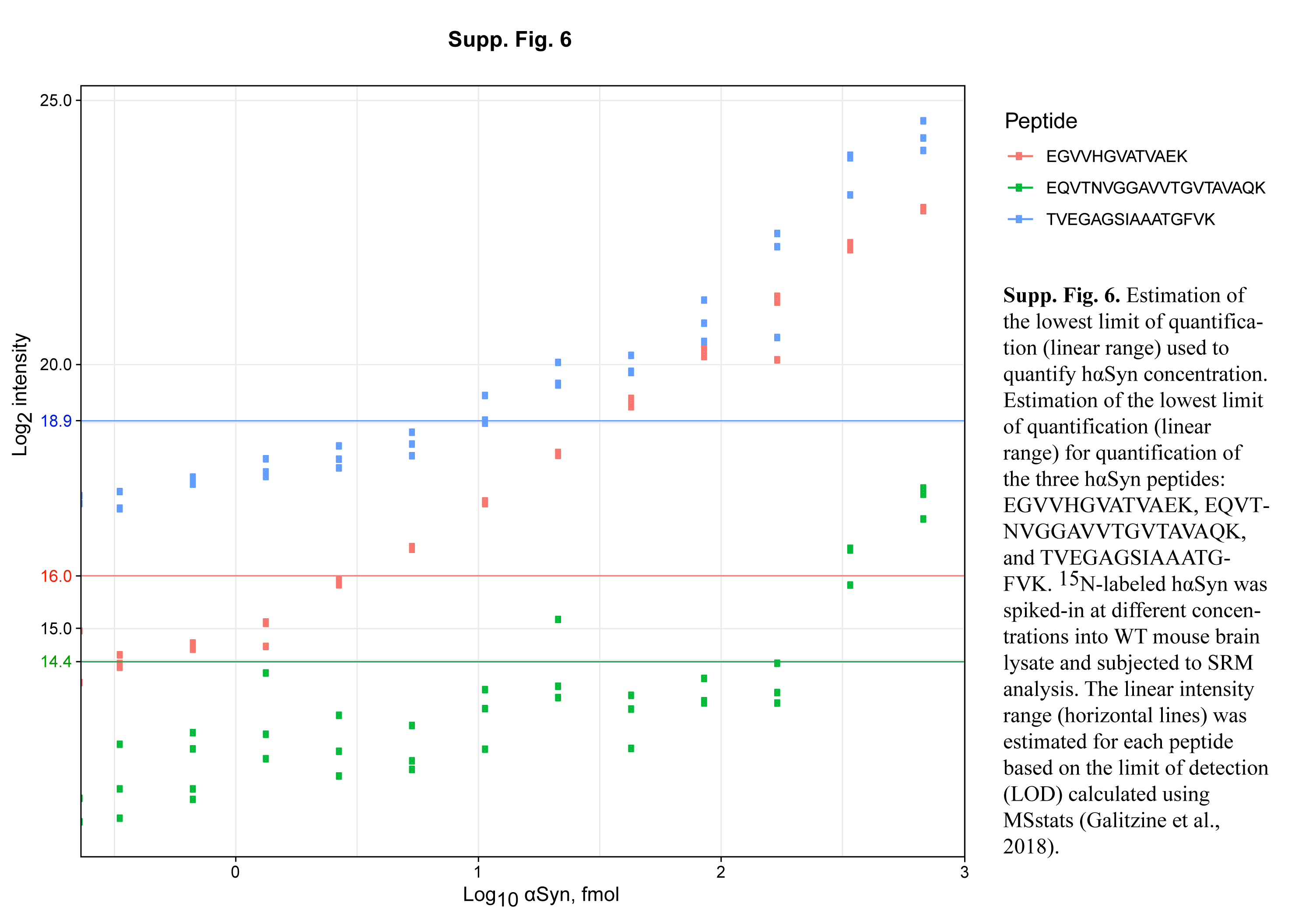

Supplement: Supplementary file 6 [file Image_6.tif]
