# Supplementary material for: Low-Expressing Synucleinopathy Mouse Models Based on Oligomer-Forming Mutations and C-Terminal Truncation of α-Synuclein
Source: Front Neurosci. 2021 Jun 17;15:643391. doi: 10.3389/fnins.2021.643391 (PMC8248494; doi:10.3389/fnins.2021.643391)
Supplement: Supplementary file 7 [file Table_1.DOCX]

Supplementary Material

# Supplementary Tables

**Supp. Table 1. Histological and biochemical analyses (associated with Figures 2B – D and 3A – D).**

| **Mouse** | **GFAP^+^ cells** | | **Iba1^+^ cells** | | **DA^‡^** | | **DOPAC^‡^** | | **HVA^‡^** | | **HVA/DA** | |
| --- | --- | --- | --- | --- | --- | --- | --- | --- | --- | --- | --- | --- |
|  | **Mean** | **± SD** | **Mean** | **± SD** | **Mean** | **± SD** | **Mean** | **± SD** | **Mean** | **± SD** | **Mean** | **± SD** |
| **WT** | 22.05 | 13.04 | 26.77 | 9.46 | 9.31 | 0.40 | 0.58 | 0.04 | 1.23 | 0.12 | 0.132 | 0.016 |
| ***hαSyn^TP^*** | 48.09 * | 13.01 | 47.31 * | 9.42 | 10.56 | 3.40 | 0.75 | 0.23 | 1.53 | 0.45 | 0.147 | 0.012 |
| ***hαSyn^Δ119^*** | 50.32 * | 12.97 | 52.64 ** | 9.38 | 9.09 | 1.88 | 0.65 | 0.17 | 1.33 | 0.34 | 0.146 | 0.014 |

SD: standard deviation; ‡: ng/mg wet tissue; comparisons to WT mice using nested 1-way ANOVA with Tukey’s multiple comparisons test (GFAP^+^, Iba1^+^) or 1-way ANOVA with Tukey’s multiple comparisons test. * p < 0.05, ** p < 0.01.

**Supp. Table 2. Vertical pole climbing abilities (associated with Fig. 4A, B).**

| **Mouse** | **Pole test** | | | | **Pole test % falling** | | | |
| --- | --- | --- | --- | --- | --- | --- | --- | --- |
|  | **1 year (s)** | | **1.5 years (s)** | | **1 year** | | **1.5 years** | |
|  | **Mean** | **± SD** | **Mean** | **± SD** | **Mean** | **± SD** | **Mean** | **± SD** |
| **WT** | 6.37 | 1.4 | 16.02 | 20.4 | 5.0 | 3.5 | 16.67 | 6.8 |
| ***hαSyn^TP^*** | 8.35 | 5.1 | 24.96 | 25.6 | 13.0 | 9.1 | 36.67 *** | 6.3 |
| ***hαSyn^Δ119^*** | 15.51 ** | 17.0 | 21.41 | 23.5 | 21.6 ** | 7.3 | 26.96 * | 8.4 |

SD: standard deviation; comparisons to WT mice using 1-way ANOVA with Tukey’s multiple comparisons test (group comparisons at 1 and 1.5 years) or repeated measures 2-way ANOVA with Bonferroni’s multiple comparisons test (% falling). * p < 0.05, ** p < 0.01, *** p < 0.001.

**Supp. Table 3. Accelerating Rotarod motor abilities (associated with Figure 4C – E).**

| **Mouse** | **Rotarod** | | | | | | | | **Rotarod** | | **Weight** | |
| --- | --- | --- | --- | --- | --- | --- | --- | --- | --- | --- | --- | --- |
|  | **Trial 1 (s)** | | **Trial 2 (s)** | | **Trial 3 (s)** | | **Trial 4 (s)** | | **Overall time (s)** | | **(g)** | |
|  | **Mean** | **± SD** | **Mean** | **± SD** | **Mean** | **± SD** | **Mean** | **± SD** | **Mean** | **± SD** | **Mean** | **± SD** |
| **WT** | 61.37 | 17.0 | 47.79 | 24.5 | 53.82 | 27.5 | 45.56 | 22.6 | 53.09 | 22.3 | 42.00 | 7.26 |
| ***hαSyn^TP^*** | 42.31 | 26.3 | 35.61 | 20.8 | 36.17 | 28.6 | 36.59 | 26.7 | 37.67 ** | 25.2 | 48.77 | 7.87 |
| ***hαSyn^Δ119^*** | 34.10 ** | 27.2 | 31.28 | 22.9 | 36.71 | 32.9 | 38.29 | 30.7 | 32.82 *** | 24.5 | 42.01 | 8.43 |

SD: standard deviation; comparisons to WT mice using repeated measures 2-way ANOVA with Bonferroni’s multiple comparisons test (trials comparisons), or 1-way ANOVA with Tukey’s multiple comparisons test (Rotarod overall time and weight comparisons). ** p < 0.01, *** p < 0.001.

**Supp. Table 4 A. CatWalk gait analysis (associated with Figure 5A, B).**

| **Mouse** | **Average speed (cm/s)** | | **Number of steps** | |
| --- | --- | --- | --- | --- |
|  | **Mean** | **± SD** | **Mean** | **± SD** |
| **WT** | 13.06 | 2.35 | 67.94 | 5.8 |
| ***hαSyn^TP^*** | 11.72 | 2.34 | 68.25 | 10.3 |
| ***hαSyn^Δ119^*** | 13.55 | 3.81 | 63.88 | 7.0 |

SD: standard deviation; comparisons to WT mice using 1-way ANOVA with Tukey’s multiple comparisons test.

**Supp. Table 4 B. CatWalk gait analysis (continued; associated with Figure 5D – F).**

| **Mouse** | **Front paws** | | | | | | **Hind paws** | | | | | |
| --- | --- | --- | --- | --- | --- | --- | --- | --- | --- | --- | --- | --- |
|  | **Paw angle to movement (°)** | | **Toe spread**  **(cm)** | | **Stand index** | | **Paw angle to movement (°)** | | **Toe spread**  **(cm)** | | **Stand index** | |
|  | **Mean** | **± SD** | **Mean** | **± SD** | **Mean** | **± SD** | **Mean** | **± SD** | **Mean** | **± SD** | **Mean** | **± SD** |
| **WT** | 26.00 | 11.2 | 0.88 | 0.05 | -2.40 | 0.47 | 12.97 | 5.5 | 1.06 | 0.06 | -4.07 | 0.91 |
| ***hαSyn^TP^*** | 32.85 | 4.8 | 0.85 | 0.05 | -2.57 | 0.61 | 12.18 | 4.2 | 1.03 | 0.08 | -3.23 | 0.76 |
| ***hαSyn^Δ119^*** | 33.27 * | 6.4 | 0.86 | 0.05 | -3.03 | 1.21 | 6.85 * | 7.2 | 0.91 *** | 0.14 | -5.95 *** | 2.08 |

SD: standard deviation; comparisons to WT mice using 1-way ANOVA with Tukey’s multiple comparisons test. * p < 0.05, *** p < 0.001.

**Supp. Table 5. Analysis of non-motor behavior (associated with Figure 6).**

| **Mouse** | **Buried food**  **latency (s)** | | **% Nesting material remaining – 1 year**  **24 h 48 h 72 h** | | | | | | **% Nesting material remaining – 1.5 years**  **24 h 48 h 72 h** | | | | | |
| --- | --- | --- | --- | --- | --- | --- | --- | --- | --- | --- | --- | --- | --- | --- |
|  | **Mean** | **± SD** | **Mean** | **± SD** | **Mean** | **± SD** | **Mean** | **± SD** | **Mean** | **± SD** | **Mean** | **± SD** | **Mean** | **± SD** |
| **WT** | 87.61 | 63.1 | 86.03 | 9.2 | 67.55 | 16.7 | 49.31 | 21.0 | 79.44 | 12.3 | 64.08 | 20.4 | 54.31 | 23.5 |
| ***hαSyn^TP^*** | 162.7 *** | 34.8 | 76.88 | 10.7 | 54.82 * | 15.6 | 35.20 * | 17.2 | 61.18 * | 15.2 | 37.78 *** | 19.6 | 24.66 *** | 19.6 |
| ***hαSyn^Δ119^*** | 167.3 *** | 28.1 | 77.17 | 15.5 | 62.97 | 17.0 | 51.70 | 21.5 | 79.72 | 13.0 | 64.19 | 19.2 | 52.71 | 22.7 |

SD: standard deviation; comparisons to WT mice using 1-way ANOVA with Tukey’s multiple comparisons test (buried food) or 2-way ANOVA with repeated measures and Bonferroni’s multiple comparisons test (nestlet-shredding). * p < 0.05, ** p < 0.01, *** p < 0.001.

**Supp. Table 6. Primer sequences of cytokines used for qPCR gene expression analysis (associated with Supp. Fig. 5).**

| **Gene symbol** | **Forward primer** | **Reverse primer** |
| --- | --- | --- |
| *TNF-α* | CCCTCACACTCAGATCATCTTCT | GCTACGACGTGGGCTACAG |
| *IL-6* | TAGTCCTTCCTACCCCAATTTCC | TTGGTCCTTAGCCACTCCTTC |
| *IL-10* | GCTCTTACTGACTGGCATGAG | CGCAGCTCTAGGAGCATGTG |
| *KC/Gro (Cxcl1)* | ACTCAAGAATGGTCGCGAGG | ACTTGGGGACACCTTTTAGCA |
| *MCP1 (Ccl2)* | CACTCACCTGCTGCTACTCA | GCTTGGTGACAAAAACTACAGC |
| *MIP-1α (Ccl3)* | CAGCCAGGTGTCATTTTCCTGA | CAGGCATTCAGTTCCAGGTCA |
| *MIP-1β (Ccl4)* | CTGTGCAAACCTAACCCCGA | AGGGTCAGAGCCCATTGGT |
